# Berry Powders as Highly Integrable Food Ingredients: Phenolic and Volatile Compounds Profiling, Comprehensive Nutrient Content Assessment and Spectroscopic Analysis

**DOI:** 10.3390/antiox15060658

**Published:** 2026-05-23

**Authors:** Miljana Djordjević, Jelena Tomić, Marijana Djordjević, Aleksandra Bajić, Jelena Živančev, Tilen Zamljen, Jerneja Jakopic

**Affiliations:** 1 Institute of Food Technology in Novi Sad, University of Novi Sad, Blvd. cara Lazara 1, 21000 Novi Sad, Serbia; marijana.djordjevic@fins.uns.ac.rs (M.D.); aleksandra.bajic@fins.uns.ac.rs (A.B.); 2 Department for Technology of Fruit Growing, Fruit Research Institute, Kralja Petra I 9, 32000 Čačak, Serbia; jtomic@institut-cacak.org; 3 Faculty of Technology Novi Sad, University of Novi Sad, Blvd. cara Lazara 1, 21000 Novi Sad, Serbia; jelena.zivancev@tf.uns.ac.rs; 4 Department of Agronomy, Biotechnical Faculty, University of Ljubljana, Jamnikarjeva 101, SI-1000 Ljubljana, Slovenia; tilen.zamljen@bf.uni-lj.si (T.Z.); jerneja.jakopic@bf.uni-lj.si (J.J.)

**Keywords:** powder, berry, seed, nutrients, fatty acids, volatiles, waste reduction, ATR-FTIR

## Abstract

The presented study aimed to fully characterise berry powders derived from raspberry, blackberry and strawberry (RB, BB, SB) as well as raspberry and blackberry seed powders (RBS, BBS) in terms of proximate composition, the individual profile of minerals, sugars, organic and fatty acids, and phenolic and volatile compounds. Additionally, testing of powders’ colour and antioxidant activity, as well as spectroscopic analysis, were also performed. Higher total and individual sugars, organic and phenolic acids, flavonols and anthocyanins content distinguished berry powders from the seed powders. Individually, RB contained significant amounts of citric and chlorogenic acids, BB was superior in cyanidin-3-*O*-glucoside and quercetin-3-*O*-rutinoside content, while SB was characterised by high sucrose, fructose, omega-3, and mineral (Ca, Mg, Fe) content. Berry seed powders exhibited remarkable TDF content, beneficial PUFA/SFA ratio, lighter colour, higher individual flavan-3-ols quantity, TPC and DPPH activity compared to berry powders. Mentioned discrepancies between berry and berry seed powders on a compositional level were also visible on ATR-FTIR spectra across all detected regions reflecting bonds attributed to cellulose, lipids, phenols and sugars. Pleasant, predominantly green, fruity and floral aromas were associated with berry powders, whilst additional herbal notes were characteristic of berry seed powders, all derived from the alcohols, aldehydes, esters and ketones as paramount volatile compounds. All examined powders can bear a nutritional claim of “high in” fibre (20.47–65.33%) and Mg (114.52–128.70 mg/100 g), enabling the design of food products packed with nutrients and bioactives while simultaneously reducing fresh fruit and fruit-processing waste.

## 1. Introduction

The consumption of berry fruits has been increasing due to growing consumer awareness of their health benefits, as they are packed with antioxidants, vitamins as bioactive compounds that contribute to the prevention of cancer, type 2 diabetes mellitus, neurodegenerative disorders, cardiovascular disease and overall well-being [1]. Despite richness in essential nutrients and bioactive compounds necessary for healthy living, berries are highly perishable [2], and together with other fruits and vegetables incur the highest post-harvest losses worldwide (>20%), excluding the retail level [3], creating environmental and economic burdens. Therefore, adequate and advanced post-harvest processing technologies are required to minimise qualitative and quantitative post-harvest losses and avoid undesirable changes in weight, colour, texture, and aroma [4], which threaten to restrict berries’ consumption, commercialization and health benefits they confer. Freezing is a widely used technique to preserve nutrients and increase the lifespan of berry fruits, and in 2022 Serbia was placed as the world’s largest supplier of this group of products.

While frozen berries are a nutritious option, their market value is relatively low when sold as basic grocery items. Thus, further processing of berries enables the transformation of quickly perishable fruits into high-quality products such as juices and beverages, jams and jellies, dried fruits, syrups, purees and concentrates, as well as fermented products like wines and vinegars, benefiting producers economically [5]. Despite significant contribution of fruit processing to the global food industry, it generates large amounts of pomace—mushy residue consisting of peels, pulp, flesh, skin, stems and seeds creating more environmental and economic challenges [6]. Fruit processing remains usually contain more bioactive compounds (flavonoids, carotenoids, anthocyanins, and polyphenolic compounds) than edible portions; represent sources of macromolecules, such as proteins, lipids, and fibres; and have antioxidant and antimicrobial properties [7]. Thus, fruit powders have emerged as functional products that are becoming increasingly popular [8] to combat mentioned challenges and support circularity likewise the achievement of Sustainable Development Goal 12 (“Responsible consumption and production”) and target 12.3, which aims to reduce “food waste”. Fruit powders can be produced from pomace, various parts of the fruit, including the pulp, peel, seeds, or even the whole fruit, providing a rich source of dietary fibre and bioactive compounds with antioxidant activity.

The potential of different wild and cultivated berry fruit powders as novel healthy ingredients was previously investigated mainly through the assessment of their antioxidant activity, phenolic and anthocyanin profiles, proximate composition, colour and antimicrobial activity [9,10,11,12]. However, corresponding results regarding commercially available berry fruit powders and powders derived from berry seeds are scarce [13,14] and predominantly related to fruits other than berries [8]. The novelty of the present study is reflected in its comprehensive approach, which investigates the powders derived from the whole fruits as well as seeds of diverse berry species through an extensive compositional analysis of the total and individual macro- and micro-constituents’ content and antioxidant activity. Additionally, powder’s volatile compounds (VOCs) are evaluated alongside ATR-FTIR analysis to assess the present functional groups and further possibilities of their interaction with other food components. With results obtained from this study, the potential for novel uses of berry fruit and seed powders as food ingredients for top-notch nutrition will be revealed, enabling diversification of berry fruit powder-enriched products, enhancement in the profitability of fruit production and processing industries and incentivizing the development of a more sustainable and circular food chain.

## 2. Materials and Methods

### 2.1. Raw Materials

Berry powders from raspberry, blackberry and strawberry (RB, BB, and SB, respectively) in the form of a commercial powder were purchased from the same producer (Jakovov, Ševarice, Serbia) where they were equally processed (air drying by condensation at 58 °C, 32 h, and subsequent milling). Raspberry and blackberry seed from cultivars ‘Willamette’ (RBS) and ‘Čačanska Bestrna’ (BBS) were obtained after juice extraction and mechanical separation based on density differences between pomace and seeds. Afterwards, seeds were dried at ambient temperature (48 h), and grinded in a coffee grinder (TSM6A017C, Bosch, Nazarje, Slovenia) during 1 min to obtain a berry seed powder (<500 μm). All berries were cultivated on domestic fields in Serbia. The obtained powders were packaged immediately in airtight bags and stored in a refrigerator until analysis.

### 2.2. Chemicals

Used chemicals were of analytical grade: methanol, Folin-Ciocalte reagent (2N), 2,2′azynobis (3-etylobenzotiazoline-6-sulfonic acid (ABTS), 2,2-difenil-1-pikrilhidrazil (DPPH), sodium acetate trihydrate and potassium chloride from Sigma-Aldrich (Schnelldorf, Germany); 6-hydroxy-2,5,7,8-tetramethylchroman-2-carboxylic acid (Trolox) and methanol from Fisher Scientific (Fair Lawn, NJ, USA); sodium carbonate and gallic acid from Carl Roth (Karlsruhe, Germany). Used standards and kits are enumerated alongside adequate methods.

### 2.3. Proximate Composition Determination

The dry matter content (%) was determined gravimetrically, by drying samples at 105 ± 0.5 °C in a convective oven to a constant mass. Afterwards, the flours’ moisture content was calculated as difference between 100 and the obtained dry matter content. The ash content was estimated according to AOAC Method 940.26 [15]. The total sugars (%) in berry powder samples were analysed by the Luff–Schoorl method while lipid content was estimated following the Soxhlet procedure. The protein content in samples was determined following the Kjeldahl method (AOAC Method 984.13-1994) [16] with the usage of conversion factor for nitrogen to crude protein-6.25.

The total dietary fibre (TDF) content was assessed by the gravimetric–enzymatic method with TDF Assay Kit (K-TDFR-200A, Megazyme, Wicklow, Ireland) according to the AOAC Method 985.29 [17].

The carbohydrate content and energetic value of samples was calculated according to Djordjević et al. [18].

### 2.4. Mineral Profile Determination

The content of macro (Na, Ca, Mg, and K) and micro elements (Fe and Zn) in berry powders was assessed by an atomic absorption spectrometer (SpectrAA 10, Varian Inc., Mulgrave Australia) following the ISO 6869:2000 method [19]. The results were expressed as an average of two conducted measurements per 100 g of flour. Additionally, for each mineral, according to the European Parliament and Council of the European Union [20], a percentage of mineral contribution was estimated as described in Djordjević et al. [21].

### 2.5. Sugars and Organic Acids Profile Determination

For analyses of individual sugars and organic acids, 0.2 g of berry powders was poured over 20 mL of double distilled water and the mixture was extracted at room temperature for 30 min on a shaker Unimax 1010 (Heidolph, Seoul, Republic of Korea) with subsequent centrifugation for 10 min at 11,292 *g* (5810 R, Eppendorf, Hamburg, Germany). The supernatant was filtered through a 0.2 μm cellulose mixed ester filter (Macherey-Nagel, Düren, Germany) and transferred to a vial for analysis.

Analysis of individual sugars was performed on a Vanquish HPLC system (Thermo Scientific, Waltham, MA, USA) equipped with refractive index (RI) detector. A 20 μL sample was injected onto a Phenomenex Rezex RCM monosaccharide column (150 × 7.8 mm; Phenomenex, Torrance, CA, USA) operating at 85 °C with a flow rate of 0.8 mL/min. Double distilled water served as the mobile phase. Sugars were identified and quantified by comparing retention times and concentrations with external standards (fructose, glucose, sucrose and sorbitol from Fluka Buchs, Switzerland). Results were expressed in mg per g of sample.

Analysis of individual organic acids was performed on a Vanquish HPLC system (Thermo Scientific, Waltham, MA, USA) equipped with ultraviolet (UV) detector operating at 210 nm. A 20 μL sample was injected onto a Phenomenex Rezex ROA Organic Acid H+ (150 × 7.8 mm; Phenomenex, Torrance, CA, USA) operating at 65 °C with a flow rate of 0.6 mL/min. Double distilled water served as the mobile phase. Organic acids were identified and quantified by comparing retention times and concentrations with external standards. For the quantification of organic acids, the following standards were used: malic acid from Merck (Darmstadt, Germany), citric and shikimic acid from Sigma-Aldrich (St. Louis, MO, USA). Results were expressed in mg per g of sample.

### 2.6. Fatty Acids Profile Determination

The individual fatty acid content in samples was determined after sample preparation involving lipid extraction, fatty acid methyl esters (FAMEs) generation and analysis by Agilent GC equipped with a flame ionisation detector FID (Agilent, 7890 Series, Santa Clara, CA, USA) as described in detail in Djordjević et al. [21]. FAMEs were identified through comparison of obtained retention times and reference standard (Supelco 37 FAME Mixture, Sigma-Aldrich, Bellefonte, PA, USA). The results were expressed in g/100 g of each identified fatty acid.

### 2.7. Phenolic Compounds’ Profile Determination

For the extraction of phenols, 0.2 g of berry powder was extracted with 5 mL of 80% methanol. The samples were sonicated for 1 h and centrifuged (5810 R, Eppendorf, Hamburg, Germany) at 7227 *g* for 7 min. The supernatants were filtered through a 0.25 µm polyamide filter (Chromafil AO-20/25, Macherey-Nagel, Dueren, Germany) and stored at −20 °C until analysis.

Identification of individual phenols was performed using tandem mass spectrometry (LTQ XL; Thermo Scientific, Waltham, MA, USA) with heated electrospray ionisation in negative ion mode. Analysis of individual phenols was carried out using a UHPLC system (Vanquish; Thermo Scientific, Waltham, MA, USA). The UHPLC-MS system settings for identification and quantification were described by Zamljen, Medic, Hudina, Veberic, and Slatnar [22].

All phenols were quantified against appropriate standards. Where no standard was available, quantification was performed using equivalents of structurally similar compounds available as standards. Specifically, all ellagic acid derivatives and ellagitannins were calculated based on ellagic acid; bis-HHDP-glucose was calculated based on gallic acid; procyanidin trimer was calculated based on procyanidin B2; quercetin-3,7-di-*O*-hexoside, quercetin-*O*-hexosyl-*O*-rutinoside, and quercetin-3-*O*-hexoside were calculated based on quercetin-3-*O*-glucoside; kaempferol glucuronide and kaempferol acetyl hexoside were calculated based on kaempferol-3-*O*-glucoside; and isorhamnetin-3-*O*-hexuronide was calculated based on isorhamnetin-3-*O*-rutinoside. Data for individual phenols are expressed as mg per kg of sample.

### 2.8. Total Phenolic Content Determination

For the determination of total phenolic content (TPC), 0.5 g of berry powder was extracted with 10 mL of 80% methanol. The samples were sonicated for 1 h and centrifuged at 9391 *g* for 5 min (Colo Instruments, Karlsruhe, Germany). The obtained extracts were used for assessing phenolic and anthocyanin content, as well as the antioxidant activity of samples. TPC of the samples was determined using the Folin–Ciocalteu assay [23] with slight modifications. An aliquot of 40 µL of extract was mixed with 3.16 mL of distilled water, 200 µL of Folin–Ciocalteu reagent (1:10 diluted), and after 8 min, 600 µL of 7.5% Na_2_CO_3_ solution was added. The mixture was made up to 4 mL and incubated in the dark for 2 h. Absorbance was measured at 765 nm using a spectrophotometer (Jenway 6300 UV-Vis, Dunmow, UK). Gallic acid was used to prepare a standard calibration curve (0–100 mg/L), and TPC was expressed as mg gallic acid equivalents (GAE) per g of sample (mg GAE/g).

### 2.9. Total Monomeric Anthocyanin Content Determination

Total monomeric anthocyanin content (TAC) was determined by the pH differential method according to Giusti and Wrolstad [24]. The absorbance of the extracts was measured at two pH values: 1.0 (potassium chloride buffer, 0.025 M) and 4.5 (sodium acetate buffer, 0.4 M). Berry powder extracts were diluted with each buffer to ensure absorbance values within the linear range of the spectrophotometer (Jenway 6300 UV-Vis, Dunmow, UK). After incubation for 15 min at room temperature, the absorbance was read at 510 and 700 nm against distilled water as a blank.

The monomeric anthocyanin pigment concentration was calculated using the following equation:

(1) $$A=\left(A_{510}-A_{700}\right)pH\;1.0-\left(A_{510}-A_{700}\right)pH\;4.5$$

(2) $$TAC\;\left(\frac{mg\;C3G}{g\;DW}\right)=\frac{A\times MW\times DF}{\varepsilon \times l}\times 1000$$

where A is the absorbance difference, A_510_ is absorbance read at 510 nm, A_700_ is absorbance read at 700 nm, MW is the molecular weight of cyanidin-3-*O*-glucoside (449.2 g/mol), DF is the dilution factor, ε is the molar absorptivity (26,900 L·mol^−1^·cm^−1^), and l is the path length of the cuvette (10 mm). Results were expressed as mg cyanidin-3-*O*-glucoside equivalents (C3G) per g of sample (mg C3G/g).

### 2.10. Antiradical Activity Determination by DPPH Assay

Antiradical activity was determined using the DPPH radical scavenging method reported by Sánchez-Moreno, Larrauri, and Saura-Calixto [25], with slight modifications. An aliquot (100 µL) of sample extract was mixed with 3.9 mL of 0.1 mM DPPH solution in methanol and vortexed. A control sample containing the same volume of methanol instead of the extract was used to measure the maximal absorbance (A_0_). After incubation in the dark for 30 min at room temperature, the absorbance was measured at 517 nm (Jenway 6300 UV-Vis, Dunmow, UK). The percentage of DPPH inhibition was calculated as

(3) $$IP\;\left(\%\right)=\frac{A_{0}-A_{sample}}{A_{0}}\times 100$$

where A_0_ is the measured absorbance of the control sample, and A_sample_ is the measured absorbance of the sample. To express the antioxidant activity in Trolox equivalents, a calibration curve was constructed using Trolox standard solutions (0–200 µM). The % inhibition values of the samples were interpolated on the Trolox calibration curve to calculate the antiradical activity as µmol Trolox equivalents per g of sample (µmol TE/g).

### 2.11. Antiradical Activity Determination by ABTS Assay

The antiradical activity of the samples was determined using the ABTS assay [26]. The ABTS radical was generated by reacting 7 mM ABTS with 2.45 mM potassium persulfate and incubating in the dark at room temperature for 12–16 h. Before the assay, the solution was diluted with methanol to an absorbance of 0.70 ± 0.02 at 734 nm. Sample extracts (30 µL) were mixed with the ABTS solution (3 mL), incubated for 6 min at room temperature, and absorbance was measured at 734 nm (Jenway 6300 UV-Vis, Dunmow, UK). Results were expressed as Trolox equivalent antioxidant capacity (TEAC) using a Trolox calibration curve (0–200 µM) and further converted to µmol Trolox equivalents per gram of sample (µmol TE/g).

### 2.12. Volatiles Profile Determination

Volatile compounds (VOCs) were extracted from the analysed samples using a DVB/CAR/PDMS fibre (Divinylbenzene/Carboxen/Polydimethylsiloxane, Supelco, Bellefonte, PA, USA). Prior to analysis, the fibre was preconditioned in the injection port of a gas chromatograph, following the manufacturer’s instructions. For each extraction, 2 g of berry flour was diluted 1:5 with ultra-pure water (Milli-Q, Merck KGaA, Darmstadt, Germany). The mixture was vortexed and centrifuged at 7012 *g* for 3 min (SL 16, Thermo Scientific, Bremen, Germany). An 8 mL aliquot of the supernatant was transferred to a 20 mL headspace vial, and 1.6 g of NaCl was added to enhance extraction efficiency by increasing the ionic strength and reducing the solubility of hydrophilic compounds in the aqueous phase.

Headspace solid-phase microextraction (HS-SPME) was conducted using an autosampler with an agitator under the following conditions. The sample was equilibrated at 50 °C for 10 min. The SPME fibre was then exposed to the sample for 40 min with periodic stirring (10 s stirring, followed by 5 s without stirring) at 500 rpm to allow VOC adsorption/absorption. After extraction, the fibre was withdrawn, placed into the gas chromatograph injector (Agilent 7890B, Santa Clara, CA, USA), and the analytes were thermally desorbed (for 3 min) and transferred directly to the analytical column. A splitless injection method was employed, and helium was used as the carrier gas at a flow rate of 1.0 mL/min.

VOCs were separated on an Agilent Technologies HP-5MS column (30 m length, 0.25 mm internal diameter, 0.25 µm film thickness; Santa Clara, CA). The temperature programme was set to start at 40 °C (for 3 min) and increase to 150 °C at a rate of 3 °C/min, then increase to 250 °C at a rate of 15 °C/min (hold time 0 min). The transfer line was maintained at 260 °C, while the ion source and quadrupole temperatures were set to 230 °C and 150 °C, respectively. SCAN mode was operated in the *m*/*z* range of 35–500 u.

Identification of VOCs was carried out by comparing the obtained mass spectra with those in the National Institute of Standards and Technology (NIST) library and Mass Hunter Workstation software (version 10.0). Retention indices (RI, normal alkane RI, non-polar column, custom temperature programme) were determined using a C8–C28 n-alkane series. Identified VOCs were confirmed by comparison of calculated RIs with the NIST database (https://webbook.nist.gov/chemistry/cas-ser/ accessed on 5 April 2024). Positive identification was considered when a good match (Match Factor ˃ 700) between the mass spectra and RI was observed. The relative abundance of individual components was expressed as the percentage of the total peak area. The data were visualised using a heat map, where each value corresponds to the contribution % of the compound to the total VOCs profile.

### 2.13. Spectroscopic Characterisation by ATR-FTIR

The berry powders spectroscopic characterisation was assessed with attenuated total reflectance (ATR) as sampling technique on Alpha II with Platinum ATR Module equipped with single reflection diamond crystal (Bruker Optics, Rosenheim, Germany) under operating conditions described in Djordjević et al. [27]. Spectra were analysed by integrated Bruker OPUS 7.0 software.

### 2.14. Colour Determination

Colour parameters of berry powders were measured by Minolta Chroma Meter CR 410 (Konica Minolta, Tokyo, Japan) in six repetitions and expressed in the CIE-L*a*b* colour space where L* indicates brightness (on a lightness-darkness scale), a* indicates hue on a green (−) to red (+) axis, and b* indicates hue on a blue (−) to yellow (+) axis. Additionally, for all samples, hue angle (h*) and chroma (C*) were identified.

### 2.15. Statistical Analysis

The one-way analysis of variance (ANOVA) (Statistica 14.0.0.15 software, TIBCO Software Inc., CA, USA) was applied to all results presented as mean values and standard deviation of at least three replicates (unless otherwise stated). The significant difference between the mean values and homogeneous groups at *p* ≤ 0.05 was established by the Duncan’s multiple range tests. Furthermore, principal component analysis (PCA) by centroid-linkage agglomerative method was applied based on correlation matrix. For determining the distance between the samples, the Euclidean measure was employed. Heatmap visualisation was performed using the RStudio (version 2025.05.1).

## 3. Results and Discussion

### 3.1. Proximate Composition and Energetic Value of Berry and Berry Seed Powders

The proximate composition of samples, along with their energetic value, is summarised in Table 1. Diverse moisture content was determined among samples ranging from 5.90 to 10.73 g/100 g, in line with previous results on commercial blackberry flour [8]. Significantly lower moisture was observed for berry seed powders (RBS and BBS) compared to the rest of the samples (*p* < 0.05, Table 1).

A similar macronutrient profile was observed across RB, BB and SB. However, significantly lower total sugars (24.92 g/100 g) and higher protein (7.28 g/100 g) contents distinguished RB from the other berry powders (Table 1). Obtained ash, lipid, protein, carbohydrate contents and energetic value for RB, BB and SB were comparable with previously reported results for freeze and convection-dried raspberry pomace [10], commercial blackberry flour [8], freeze-dried blackberry [28] and freeze-dried strawberries [29]. Furthermore, the RBS and BBS macronutrient profiles were diverse compared to the berry powder samples, delivering higher lipid (12.16 and 16.28 g/100 g, respectively) and TDF content (65.33 and 58.25 g/100 g, respectively), followed by remarkably lower total sugars content (Table 1). Differences in most of the observed macronutrients between berry seed powders were not statistically significant (Table 1), except protein and lipid content, and consequently, the energetic value, which were significantly higher in BBS compared to RBS (*p* < 0.05). A previous study reported lower lipid and higher protein and TDF content in defatted blackberry seeds [30] compared to BBS, whilst results on RBS macronutrient content were predominantly congruent with previous studies [5].

Mentioned distribution of macronutrients in berry and berry seed powders was expected considering the sugar accumulation in the vacuoles of fruit tissue, essential for fruit development and ripening [31], as well as seeds’ predominant accumulation of storage compounds (lipids and proteins), and precursors for hormonal and secondary metabolites for future germination and growth [32]. Above twofold higher levels of TDF found in RBS and BBS when compared to corresponding berry powders (Table 1) could be explained by the dominance of the insoluble fibre fraction, consisting of structural polysaccharides, predominantly cellulose, that support seed coat rigidity, forming a physical barrier that protects the seed and ensures its viability for the next generation [32]. Higher TDF content than obtained herein was reported for raspberry, blackberry and strawberry pomaces ranging between 62 and 80 g/100 g [33], and likewise for different berry pomaces, ranging between 29.24 and 61.16 g/100 g [9]. Still, all samples had TDF content greater than 6 g/100 g and can bear the nutritional claim “high in fibre” according to the EC regulation No 1924/2006 [34].

**Table 1:** Proximate composition and mineral profile of examined berry and berry seed powders.

	RB	RBS	BB	BBS	SB
**Proximate composition [%]**					
Moisture	9.82 ± 0.61 ^a^	5.90 ± 0.39 ^c^	8.77 ± 0.58 ^b^	6.72 ± 0.44 ^c^	10.73 ± 0.71 ^a^
Dry matter	90.18 ± 0.61 ^c^	94.10 ± 0.39 ^a^	91.23 ± 0.58 ^b^	93.28 ± 0.44 ^a^	89.27 ± 0.71 ^c^
Ash	2.59 ± 0.2 ^b^	1.35 ± 0.1 ^d^	2.12 ± 0.16 ^c^	1.50 ± 0.11 ^d^	3.12 ± 0.24 ^a^
Lipids	3.96 ± 0.64 ^c^	12.16 ± 1.97 ^b^	5.07 ± 0.82 ^c^	16.28 ± 2.63 ^a^	2.87 ± 0.46 ^c^
Total sugars	24.92 ± 5.3 ^b^	1.82 ± 0.39 ^c^	41.59 ± 8.85 ^a^	2.39 ± 0.51 ^c^	43.36 ± 9.23 ^a^
Proteins	7.28 ± 0.37 ^b^	7.08 ± 0.36 ^b^^c^	6.27 ± 0.32 ^d^	9.16 ± 0.47 ^a^	6.42 ± 0.33 ^cd^
Carbohydrates	48.80 ± 4.3 ^a^	8.18 ± 3.82 ^b^	52.34 ± 4.17 ^a^	8.09 ± 3.65 ^b^	56.39 ± 3.58 ^a^
TDF	* 27.55 ± 2.48 ^b^	65.33 ± 5.88 ^a^	25.43 ± 2.3 ^b^	58.25 ± 5.24 ^a^	20.47 ± 1.84 ^b^
Energy [kJ/100 g]	1320.28 ± 23.29 ^c^	1231.98 ± 21.85 ^d^	1387.40 ± 16.78 ^a^	1361.61 ± 3.94 ^ab^	1337.72 ± 23.53 ^bc^
Energy [kcal/100 g]	315.06 ± 5.00 ^b^	301.14 ± 3.87 ^c^	330.93 ± 3.44 ^a^	332.02 ± 0.46 ^a^	318.01 ± 5.18 ^b^
**Mineral profile [mg/100 g]**					
Ca	89.71 ± 0.73 ^d^	91.51 ± 2.37 ^d^	**126.45 ± 0.19** ^b^	109.04 ± 0.12 ^c^	**152.20 ± 1.48** ^a^
K	** **593.14 ± 2.75** ^b^	175.45 ± 0.21 ^e^	296.56 ± 0.05 ^c^	220.91 ± 2.90 ^d^	*** **758.59 ± 19.58** ^a^
Mg	**117.54 ± 1.96** ^b^	**114.52 ± 4.82** ^b^	**128.70 ± 1.13** ^a^	**114.67 ± 3.24** ^b^	**119.07 ± 1.81** ^b^
Na	28.64 ± 1.62 ^c^	14.11 ± 0.43 ^e^	18.91 ± 0.25 ^d^	39.98 ± 0.38 ^b^	54.41 ± 3.22 ^a^
Fe	**4.02 ± 0.11** ^d^	**4.44 ± 0.02** ^c^	**7.04 ± 0.09** ^a^	**4.97 ± 0.25** ^b^	**4.89 ± 0.14** ^b^
Zn	**1.98 ± 0.03** ^b^	**1.63 ± 0.05** ^d^	**1.77 ± 0.14** ^c^	**2.34 ± 0.04** ^a^	**1.84 ± 0.02** ^c^

RB—commercial raspberry powder, RBS—raspberry seed powder, BB—commercial blackberry powder, BBS—blackberry seed powder, SB—commercial strawberry powder, TDF—total dietary fibre content. Carbohydrates were calculated by difference: 100 − (moisture + ash + protein + lipid + total dietary fibre). Means in the rows for each determined parameter followed by different letters are significantly different (*p* < 0.05), according to Duncan’s multiple range test. * Underlined values—“High in fibre” according to regulation (EC) No 1924/2006 [34]. ** Bold values—“Source of mineral” according to regulation (EC) No 1169/2011 [34] and (EC) No 1924/2006 [34]. *** Underlined bold values—“High in mineral” according to regulation (EC) No 1169/2011 and (EC) No 1924/2006.

### 3.2. Mineral Profile of Berry and Berry Seed Powders

The most abundant minerals in samples were K, followed by Ca and Mg, whilst Zn content was the lowest (Table 1). The reported descending order in mineral quantity is consistent with that reported in fresh strawberry and raspberry [35], freeze-dried raspberry powder [36], spray-dried strawberry powder [37], commercial blackberry flour [8], and freeze-dried blackberry powder [28]. SB was characterised by the highest Ca (152.20 mg/100 g), K (758.59 mg/100 g) and Na (54.41 mg/100 g) content, whilst BB had the highest Mg (128.70 mg/100 g) and Fe (7.04 mg/100 g) content, and for BBS the highest content of Zn was recorded (2.34 mg/100 g, Table 1).

Significant differences in Ca, K, Na, and Fe contents were observed among RB, BB and SB, whilst the quantity of Mg and Zn significantly distinguished only for BB and RB, respectively (*p* ˃ 0.05, Table 1). The obtained results also differed from those previously published in terms of higher Mg and Zn; lower Ca, K, and Fe content in RB compared to raspberry freeze-dried powder [36]; higher Ca, lower K, Mg, Fe and Zn amounts in SB compared to spray dried strawberry powder [37]; and higher Mg, Na, Fe, and Zn, but lower K and Ca content in BB than previously reported for commercial blackberry flour [8] and freeze-dried blackberry powder [28]. Differentiation among berry and berry seed powders was most noticeable in the content of K and Mg, with corresponding contents higher in RB, BB and SB, while such a clear trend was not observed for other investigated minerals (Table 1). Significantly higher content of all examined minerals was found in BBS compared to RBS (*p* ˃ 0.05, Table 1). Still, RBS exhibited higher Ca, K and Mg, but lower Fe and Zn content than reported for raspberry seeds before oil extraction [38].

Nevertheless, calculated DRI value for the investigated minerals showed that all samples can be considered as “high in Mg” and a “source of Zn” as they provide 30% and 15% of corresponding minerals in daily intake, in accordance with EC regulation No 1924/2006 [34] (Table 1). Additionally, all samples could be categorised as “high in Fe”, except for RB, which qualifies as a “source of” this mineral. SB is “high in K” and a “source of Ca”, whilst BB and RB are considered as sources of Ca and K, respectively, according to the same regulation. Greater mineral content in berry powder samples could be related to the presence of dietary fibres with high affinity for mineral binding in berry powders [39]. Minerals are involved in many diverse metabolic functions in the body, and their intake is deficient considering increasing consumption of cereal-based and refined food products [37]. Thus, incorporating berry powders with exceptional mineral profiles into staple foods, such as bread, and gluten-free foods, can be a straightforward strategy for achieving a mineral-enriched diet.

All mentioned discrepancies in macronutrients and mineral profile and content among investigated powders and literature data could be a consequence of diversity in fruit species, variety and genotype, agricultural practices, berry ripeness, postharvest treatments, fruit processing and drying conditions to obtain powder/flour, as already emphasised by other authors [5,8].

### 3.3. Sugars and Organic Acids Composition in Berry and Berry Seed Powders

In berry powders (RB, BB and SB), fructose was present in the highest amount, followed by glucose and significantly lower levels of sucrose and sorbitol (Table 2). The 30% higher level of total sugars was detected in SB compared to RB and BB. No significant difference in the sum of sugars was detected between RB and BB. The higher levels of sugars found in SB could be attributed to the higher sugar content of fresh strawberry fruits compared to raspberry and blackberry fruits [40]. Even though fructose has higher sweetness intensity than glucose and sucrose, in fresh fruit, the total sugar content generally represents a better indicator of consumer taste acceptability than fructose content.

The fructose, glucose and sum of sugars in RB were comparable to freeze-dried raspberry powder reported by Marino et al. [36], whilst sucrose content detected herein was lower. Still, the highest sucrose content is observed in RB (Table 2), and can be ascribed to the intrinsic characteristics of the raw material [41]. On the other hand, the sum of sugars in RBS and BBS was approximately twenty and ten times lower, respectively, compared to the sum of sugars in RB and BB. However, discrepancies compared to results of total sugars determined by the Luff–Schoorl method (Table 1) can be ascribed to the detection of different sugar fractions (primarily reducing ones), fluctuations in reaction conditions, as well as methodological limitations regarding stoichiometry and linearity, which may have an underestimated influence on the result. Although the content of all individual and total sugars in BBS was twice as high as in RBS, the sugar profile of both powders was similar.

Glucose and sucrose were the dominant sugars, and sorbitol was not detected. Krstić et al. [42] confirmed that blackberry, strawberry, black currant, raspberry and blueberry seed samples were differentiated according to lipid (di- and triglycerides, and sterol esters) and free acid profiles, while only black currant and strawberry seed samples showed specific sugar profiles. Recently, the total sugar level in raspberry seeds of cultivar ‘Polka’ before oil extraction was analysed using a method that detects the total amount of sugar (2.8%) but fails to specify the content of individual sugars [38]. The reported result was lower compared to the sum of individual sugars identified herein for RBS. However, similar individual sugar composition and content were reported by Hurtado-Romero et al. [9], except in strawberry, raspberry, blueberry, and blackberry bagasse.

Nevertheless, available studies report only the total organic acid content of different fruit powders, with no detailed information on individual organic acids. Therefore, the content of organic acids in raspberry and blackberry seeds was studied for the first time. Previous research on berry seeds has been mainly focused on the individual content of biologically active compounds because seeds have a higher concentration of certain phytochemicals than other parts of the fruit [43]. In RB, SB, and BBS, citric acid is the most abundant organic acid, accounting for 60–70% of the total acidity, whereas the proportion of malic acid is considerably lower, ranging from 30 to 40%. On the other hand, comparable levels of citric and malic acids were measured in RBS and BB (Table 2). Shikimic acid was present in much smaller amounts compared to citric and malic acids, regardless of the powder sample. Considering total acidity, previous study on various commercial fruit flours undertaken by [8] estimates the acidity of blackberry flour (8.1%) in an amount comparable to BB. On the other hand, Marino et al. [36], who used lyophilisation to obtain raspberry fruit powder, reported a threefold lower content of total acids (5.8%) compared to RB (180.78 mg/g ~18%, Table 2). Given that variability in organic acid metabolism has been reported in many fruit species [44], and that numerous genetic studies have shown that the accumulation of organic acids (e.g., malic acid) is controlled by genes, with differences not only between species but also between cultivars [45], the observed differences in organic acid content in investigated samples can be explained by the different levels of these acids in the fresh fruits from which the powders were obtained.

### 3.4. Fatty Acid Profile of Berry and Berry Seed Powders

Fourteen fatty acids were detected in the investigated powders, with saturated fatty acids (SFA) most abundant in number and polyunsaturated fatty acids (PUFA) most abundant in content (Table 2), consistent with previous studies [5,13,35,36,46].

The total SFA content was higher in berry powders, the highest in SB (14.20 g/100 g total fatty acids, Table 2), compared to berry seed powders. Conversely, berry seed powders exhibited higher total PUFA content, the highest in RBS (79.80 g/100 g total fatty acids, Table 2), than other berry powders investigated. When considering total monounsaturated fatty acids (MUFA), BBS had the highest content (17.40 g/100 g total fatty acids, Table 2), followed by RB (16.20 g/100 g total fatty acids, Table 2). Up to two-fold higher content of total PUFA was observed in RB and BB compared to previously published results on freeze-dried raspberry and blackberry, with comparable total MUFA and significantly lower total SFA contents [46]. Similarly, RBS total PUFA content was five times higher compared to cold-pressed seed flours of two raspberry varieties, whilst lower total MUFA and SFA contents were obtained [13]. Observed discrepancies could be explained by different extraction and detection methods applied, and likewise by fatty acid content dependence on fruit species and variety.

Regarding individual fatty acids, the results followed the same trend as for the total ones, with linoleic and α-linolenic acids being the most abundant as PUFAs, oleic acid as MUFA, and palmitic acid belonging to the SFA group (Table 2). Higher contents of palmitic and α-linolenic acid (omega-3) were detected in berry powders compared to berry seed powders, while the opposite was observed for linoleic acid (omega-6) (Table 2). SB was distinguished from the other powder samples with the highest palmitic (9.90 g/100 g total fatty acids) and α-linolenic acid content (27.30 g/100 g total fatty acids), while oleic (17.40 g/100 g total fatty acids) and linoleic acids (62.80 g/100 g total fatty acids) were the most abundant in BBS (Table 2). The contents of corresponding fatty acids in RB were in line with those of freeze-dried raspberry [36]. Higher oleic and linoleic acid levels, and lower levels of palmitic and α-linolenic acids were observed in BB compared to freeze-dried blackberry [46]. Limited results are available on the fatty acid composition of strawberries, predominantly related to fruits and derived seed oils [35,47]. Corresponding fatty acids were also the main ones found in strawberry seed oil, and while the content of oleic and linoleic acids was comparable to SB, higher amounts of palmitic and lower amounts of α-linolenic acid were found in SB compared to strawberry seed oil [47].

BBS and BB were characterised by high linoleic acid (omega-6) contents (62.80 and 61.40 g/100 g total fatty acids, respectively), while the higher content of α-linolenic acid (omega-3) was detected in RB and RBS (25.70 and 25.10 g/100 g total fatty acids, respectively) consistent with their prevalence in blackberry and raspberry seed oils [48]. Corresponding contents reflected on the omega-6/omega-3 ratio ranging from 1.57 for SB to 5.33 for BBS. Since the omega-6/omega-3 ratio considered optimal according to WHO should not exceed 4.0 [5,46], SB, RB and RBS can be claimed as beneficial for human nutrition. Nevertheless, the prevalence of PUFAs is evident, and the obtained PUFA/SFA ratios for all investigated powders were higher than 0.5, as recommended by the WHO [5,46], ranging from 4.93 (SB) up to 13.3 (RBS), expressing an elevated amount, which is an indicator of the nutritional quality of the fat in the diet [36].

### 3.5. Phenolic Profile of Berry and Berry Seed Powders

**Figure 1:**
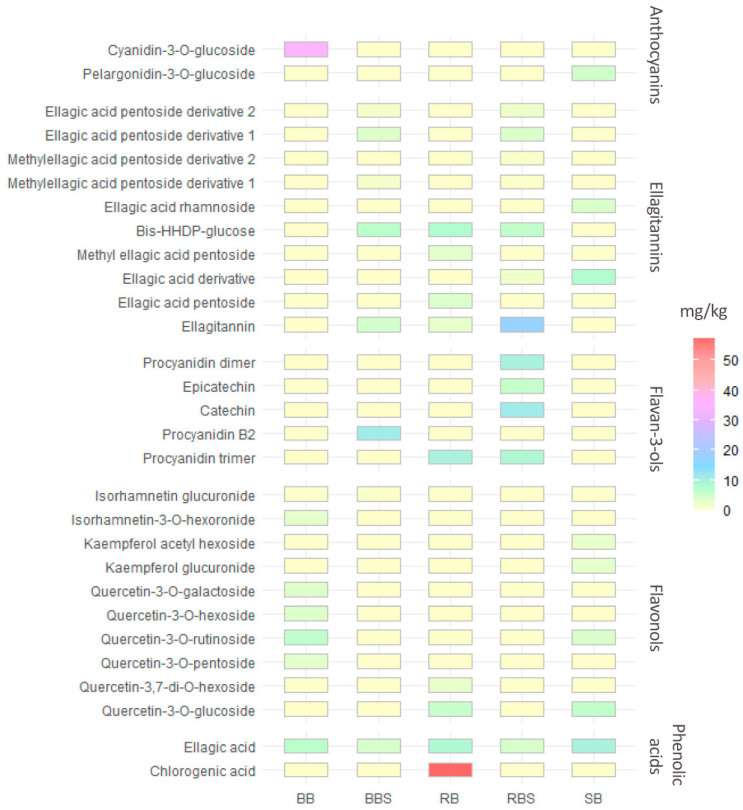
The heatmap of the concentration (mg/kg) of anthocyanins, ellagitannins, flavan-3-ols, flavonols and phenolic acids alongside corresponding individual compounds in berry and berry seed powders. The colour key represents differences in the concentration of detected compounds. Based on Supplementary Table S1. RB—commercial raspberry powder, RBS—raspberry seed powder, BB—commercial blackberry powder, BBS—blackberry seed powder, SB—commercial strawberry powder.

The conducted UHPLC-MS analysis revealed differences among powders regarding the individual phenolic profile and content, while dominant sub-classes were hydroxycinnamic acids, hydroxybenzoic acids (mainly ellagitannins), flavonols, flavan-3-ols and anthocyanins (Figure 1).

Considering berry powders, the presence of twenty phenolic compounds was confirmed, including two phenolic acids, six ellagitannins, nine flavonols, one flavan-3-ol, and two anthocyanins (Figure 1). The only common compound for all powders was ellagic acid (sub-class of hydroxybenzoic acid), where its content in berry powders ranged between 6.74 and 9.57 mg/kg and was higher compared to berry seed powders (~4.2 mg/kg) (Figure 1, Supplementary Table S1).

RB was distinguished as the only powder with detected chlorogenic acid in a significant amount (58.18 mg/kg; Supplementary Table S1), followed by bis-HHDP-glucose, ellagic acid pentoside, methyl ellagic acid pentoside, and ellagitannin. Similarly, the presence of ellagic acid and its derivatives was previously reported for lyophilised raspberry pomace [11] and defrosted wild raspberry [49]. Furthermore, the presence of ellagic acid derivative and ellagic acid rhamnoside differentiates SB from RB and BB. Based on the total phenolic acids content, RB significantly stands out, followed by SB, while minimal content was recorded in BB (ellagic acid-6.74 mg/kg; Supplementary Table S1). Nevertheless, great versatility was observed in the flavonols composition, primarily in BB with five detected flavonols, followed by SB with four and RB with two detected corresponding compounds.

Unlike other powders, BB contained quercetin-3-*O*-rutinoside, quercetin-3-*O*-hexoside, quercetin-3-*O*-galactoside and quercetin-3-*O*-pentoside in descending amounts (Figure 1, Supplementary Table S1). The quercetin-3-*O*-rutinoside content was the highest in BB, consistent with previous research [50,51], while lower in SB, and not detected in RB. Furthermore, the quercetin-3-*O*-glucoside content was maximal in SB, followed by RB. A distinction was also observed for SB, as the only powder with detected kaempferol glucuronide and kaempferol acetyl hexoside. Regarding the total flavonols content, the obtained values were in descending order: BB > SB > RB (Supplementary Table S1). The absence of flavan-3-ols was visible in berry powders, since the procyanidin trimer was only detected in RB. Conversely, from the sub-class of anthocyanins, pelargonidin-3-*O*-glucoside was noticed only in SB in line with findings of Hurtado-Romero et al. [9] and Vega et al. [12], while cyanidin-3-*O*-glucoside emerged as a major compound in BB (34.72 mg/kg; Supplementary Table S1) in accordance with the literature [9,50,51], bringing BB to the forefront as a source of anthocyanins.

The berry seed powders were characterised by the presence of 13 phenolic compounds, namely eight elagitannins including several derivatives, one flavonol, five flavan-3-ols, and without detected anthocyanins (Figure 1, Supplementary Table S1). Besides ellagic acid and ellagitannins as the most prominent regarding content, other shared compounds in RBS and BBS were bis-HHDP-glucose, methylellagic acid pentoside derivative 1, ellagic acid pentoside derivative 1 and ellagic acid pentoside derivative 2 (Supplementary Table S1) and the presence of enumerated derivatives distinguished corresponding powders from the berry powders. Furthermore, RBS was the most prominent regarding the ellagitannin content (17.79 mg/kg) compared to the rest of the powders. Similarly, BBS stands out as the only powder with detected isorhamnetin glucuronide (0.57 mg/kg) from the flavonols sub-class. The flavan-3-ols as sub-class were the most assorted in RBS where, in descending amount, catechin, procyanidin dimer, procyanidin trimer and epicatechin were disclosed, consistent with the results of Mannino et al. [52]. Nevertheless, BBS gave its contribution as the only sample with detected procyanidin B2. Although similar regarding the detected amount of catechin and procyanidin B2 in RBS and BBS (Supplementary Table S1), respectively, RBS, as the most abundant in flavan-3-ols yielded the highest total flavan-3-ol amount regardless of sample.

By content, the dominant individual compounds were chlorogenic acid in RB, cyanidin-3-*O*-glucoside in BB, ellagic acid in SB, ellagitannin in RBS and procyanidin B2 in BBS (Supplementary Table S1). Considering the total phenolic compounds content, RB emerged as the most favourable (101.78 mg/kg) while lower amounts were observed for the rest of the powders in descending order. RBS > BB > SB > BBS. A major contribution to the quantification was delivered by phenolic acids in RB, ellagitannins in BBS and SB, and likewise flavan-3-ols and anthocyanin in RBS and BB, respectively (Supplementary Table S1). However, in order to elucidate the physiological relevance of the detected phenolic compounds, further studies addressing their bioaccessibility and bioavailability need to be conducted.

### 3.6. Antioxidant and Antiradical Activity of Berry and Berry Seed Powders

Results obtained regarding TPC, TAC, and antiradical activity of berry and berry seed powders are summarised in Table 3.

Considering TPC, a significant distinction was observed between berry powders (48.05–58.22 mg GAE/g) and berry seed powders (75.26–77.16 mg GAE/g) since RBS and BBS showed 25–35% greater TPC compared to RB, BB and SB, assuming the secondary metabolites precursors accumulation in seeds [32]. The corresponding data on berry seed powders are limited; nevertheless, the RBS exhibited significantly greater TPC than reported previously [13]. The obtained results for RB and BB were in line with results of Krupa-Kozak et al. [51] on lyophilised berry powders, while lower values were reported by Hurtado-Romero et al. [9], Pecyna et al. [10] and Różyło et al. [11] for corresponding pomace powders. Nevertheless, the only available data related to TPC of commercial blackberry flour [8] was comparable to BB in the present study. Furthermore, detected TPC of SB herein was significantly higher compared to previously detected values by Hurtado-Romero et al. [9] in lyophilised strawberry pomace. Nevertheless, the observed discrepancy in TPC and sum of identified individual polyphenols by HPLC can be primarily ascribed to the structure–activity relationships between phenolic compounds classes and the Folin–Ciocalteu reagent and hence the particular reaction mechanism involved single electron transfer (SET) [53].

The TAC was not observed in berry seed powders in accordance with Parry et al. [13], but rather in berry powders (RB, BB and SB). The highest amounts observed were for SB and BB (0.65 and 0.63 mg C3G/g, respectively) while being significantly reduced in RB (Table 3). The opposite sequence was reported by Hurtado-Romero et al. [9] where the order was blackberry, raspberry and strawberry pomace with all TAC values higher compared to those in the present study. Nevertheless, similar TAC was detected in freeze-dried raspberry pomace [10], while lower values were reported in fresh raspberries and blackberries [46], but were also higher for fresh wild strawberry by QUENCHER methodology [12].

The ability of scavenging DPPH radicals was the most prominent for RBS and BBS (229.98 and 219.45 μmol TE/g), while significant reduction in that ability was observed for RB and BB, and especially SB (141.86 μmol TE/g, Table 3). Nevertheless, RB was highlighted among berry powders concerning DPPH as previously reported [46], considering that there are no comparable data on berry seed powders.

A similar trend was observed for the ABTS assay where BBS was established as the most efficient in ABTS radical scavenging (21.26 μmol TE/g, Table 3), followed by RBS, and subsequently BB, and RB, while SB activity in this respect was the lowest (Table 3). Although comparable data on berry seed powders are lacking, the obtained results for RB and BB are consistent with the results of Krupa-Kozak et al. [51], where strong scavenging ability was determined for lyophilized raspberry and blackberry. However, studies reporting higher ABTS values for wild raspberry and pomaces from different blackberry varieties also exist [49,50]. Nevertheless, previous findings also emphasised blackberry’s antioxidant role in ABTS radical scavenging, associating it with particular anthocyanin [46], which was detected in BS as well (Figure 1, Supplementary Table S1). In general, the results from antiradical assays reflect the determined greater TPC in RBS and BBS compared to the remaining berry powders.

All discrepancies in the obtained and existing results are a consequence of multiple factors: berry variety, growing conditions, harvest time [51], applied extraction technique and conditions, as well as the conducted assay methodology due to lack of standardisation for these methods [54]. Additionally, the examined samples herein underwent thermal and milling treatment, which also influences antioxidant activity in a certain way.

### 3.7. Volatile Compounds Profile of Berry and Berry Seed Powders

**Figure 2:**
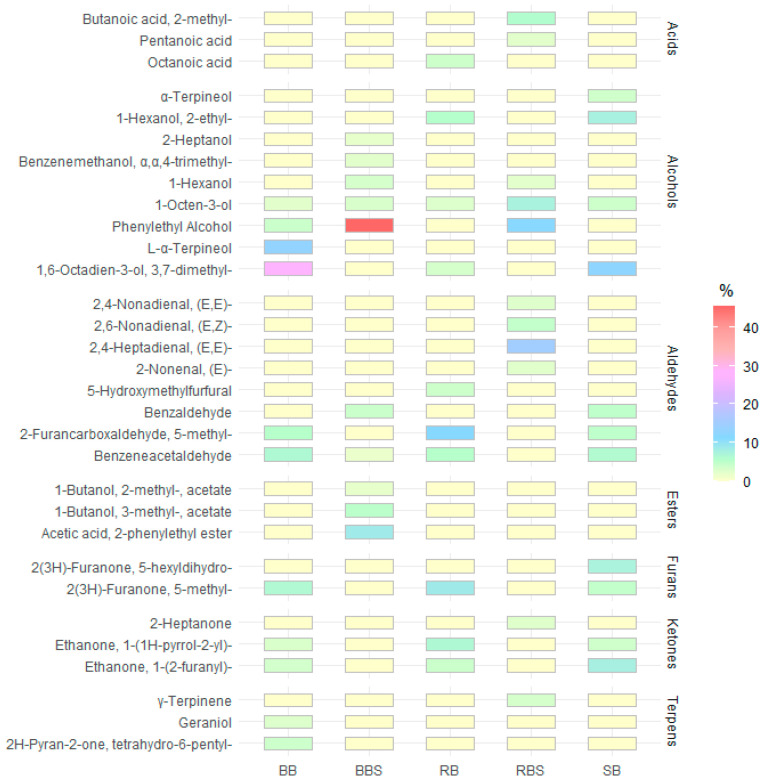
The heatmap presenting the share (%) of the most present individual volatile compounds and volatile compounds classes detected in berry and berry seed powders. The colour key represents differences in the share of detected compounds. Based on Supplementary Table S2. RB—commercial raspberry powder, RBS—raspberry seed powder, BB—commercial blackberry powder, BBS—blackberry seed powder, SB—commercial strawberry powder.

The recognisable and exceptional flavour of berry fruits is a combination of diverse VOCs [55] with a potential to be retained and imparted to fruit-derived powders and product thereof. A total of 156 VOCs were identified in berry and berry seed powders belonging to almost all chemical classes with esters (42), aldehydes (33), alcohols (25) and ketones (23) as the most represented (Figure 2, Supplementary Table S2).

Most of the identified VOCs align with the known aroma components of berry fruits, delivering their recognisable fruity (esters), floral (terpenes), sweet (furanones), and green (aldehydes) aroma [55,56]. Considering the individual compounds, 2-hexenal, (E)-, heptanal, hexanoic acid, methyl ester, 2-heptenal, (Z)-, 5-hepten-2-one, 6-methyl-, o-cymene, 2-octenal, (E)-, acetophenone, 1,6-octadien-3-ol, 3,7-dimethyl-, nonanal, 2-nonenal, (E)-, 2(3H)-furanone, dihydro-5-pentyl-, n-hexadecanoic acid were present in all powder samples with benzaldehyde, 1-octen-3-ol, benzeneacetaldehyde, and phenylethyl alcohol as the most abundant across all samples (Figure 2).

As regards berry powders, 1,6-octadien-3-ol, 3,7-dimethyl- and 2-furancarboxaldehyde, 5-methyl- were VOCs with the highest content in BB (27.61%), SB (12.37%) and RB (11.54%), respectively, belonging to the alcohol and aldehyde classes (Figure 2). In BB, four alcohols (1,6-octadien-3-ol, 3,7-dimethyl-, L-.alpha.-terpineol, phenylethyl alcohol, and 1-octen-3-ol), two aldehydes (benzeneacetaldehyde and 2-furancarboxaldehyde, 5-methyl-), two terpenes (2H-pyran-2-one, tetrahydro-6-pentyl- and geraniol), two ketones (ethanone, 1-(2-furanyl)- and ethanone, 1-(1H-pyrrol-2-yl)-), and one furan (2(3H)-furanone, 5-methyl-) were found as major VOCs with quantities ranging from 2.37 up to 27.61% (Figure 2, Supplementary Table S2). Alcohols were the most abundant compounds in BB (52.94%), in accordance with previous studies on wild blackberry [57]. α-terpineol, phenylethyl alcohol, and geraniol, found in higher quantities in BB, were also identified as major volatiles in fresh blackberries [55,56]. β-Ionone, defined as one of the most potent odorants of fresh blackberries [55], had the lowest content in BB (0.08%) compared to berry powders investigated in this study. Additionally, 2-heptanol, recognised as responsible for the herbaceous and citrus flavour in fresh berries [58], was identified in a quantity of 1.00% only in BB among berry powders (Figure 2, Supplementary Table S2). To date, studies investigating the content and profile of VOCs in blackberries dried by different drying techniques are scarce. However, according to the results obtained, the investigated BB can be characterised by floral, fruity, and green aroma due to the presence of alcohols, terpenes, and ketones. Predominant VOCs in RB were three aldehydes (2-furancarboxaldehyde, 5-methyl-, benzeneacetaldehyde, and 5-hydroxymethylfurfural), three alcohols (1-Hexanol, 2-ethyl-, 1,6-octadien-3-ol, 3,7-dimethyl-, and 1-octen-3-ol), two ketones (ethanone, 1-(1H-pyrrol-2-yl)- and ethanone, 1-(2-furanyl)-), one furan (2(3H)-furanone, 5-methyl-) and one acid (octanoic acid) with contents ranging from 2.89 to 11.54% (Figure 2, Supplementary Table S2). α-ionone and β-ionone, which are of particular importance as VOCs in the fresh red raspberry aroma [55], were also identified in RB in quantities of 1.14 and 1.78%, respectively. However, a low content (0.11%) of a single compound characterised by raspberry odour (2-Butanone, 4-(4-hydroxyphenyl)-) and referred to as “raspberry ketone” [55] was detected in RB (Figure 2, Supplementary Table S2). When thermally processed, VOCs of raspberries undergo changes which include decline in esters and increase in aldehydes and ketones [59], as well as the reduction in the main carriers of raspberry aroma α-ionone, β-ionone and raspberry ketone, thus weakening the floral, raspberry-like and violet-like note of RB powder aroma [60]. Four alcohols (1,6-octadien-3-ol, 3,7-dimethyl-, 1-hexanol, 2-ethyl-, 1-octen-3-ol, and α-terpineol), three aldehydes (benzeneacetaldehyde, 2-furancarboxaldehyde, 5-methyl-, and benzaldehyde), two ketones (ethanone, 1-(2-furanyl)- and ethanone, 1-(1H-pyrrol-2-yl)-), and two furans (2(3H)-furanone, 5-hexyldihydro- and 2(3H)-furanone, 5-methyl-) were the most abundant VOCs in SB with contents ranging from 3.85 to 12.37% (Figure 2, Supplementary Table S2). The presence of aldehydes and ketones in higher quantities is in line with previously published results on strawberries and derived products dried by diverse drying techniques [61], giving a more green, fruity, honey and almond-like note to the aroma of SB [62].

When considering berry seed powders, phenylethyl alcohol and 2,4-heptadienal, (E,E)- as aldehyde had the highest contents in BBS (45.33%) and RBS (14.87%), respectively (Figure 2, Supplementary Table S2). The content of prevailing VOCs in BBS ranged from 1.72 up to 45.33% and included five alcohols (phenylethyl alcohol, 1-hexanol, 1-octen-3-ol, benzenemethanol, α,α,4-trimethyl-, and 2-heptanol), three esters (acetic acid, 2-phenylethyl ester, 1-butanol, 3-methyl-, acetate, and 1-butanol, 2-methyl-, acetate), and two aldehydes (benzaldehyde and benzeneacetaldehyde) (Figure 2, Supplementary Table S2). It is assumed that the presence of particular esters among the most abundant compounds in BBS is related to their synthesis as maturation of blackberry progresses and is mainly associated with its seed [58]. Although there are no available data on content and profile of VOCs in blackberry seed and derived powder, the presence of 1-hexanol and 2-heptanol in higher quantities in BBS is consistent with results obtained for blackberry pomace from different cultivars [50]. Major VOCs identified in RBS were four aldehydes (2,4-heptadienal, (E,E)-, 2,6-nonadienal, (E,Z)-, 2,4-nonadienal, (E,E)-, and 2-nonenal, (E)-), three alcohols (phenylethyl alcohol, 1-octen-3-ol, and 1-hexanol), two acids (butanoic acid, 2-methyl- and pentanoic acid), one terpene (γ-terpinene), and one ketone (2-heptanone) with contents ranging from 2.32 to 14.87% (Figure 2, Supplementary Table S2). Paucity of studies investigated VOCs content and profile in raspberry seed, whilst there are available results for raspberry seed oil [63]. Interestingly, 2,4-heptadienal, (E,E)- found as the most abundant in RBS, was not detected in raspberry seed oil [63]. It is assumed that an increase in VOCs (aliphatic alcohols, aldehydes, ketones, acids, and esters) in a food product containing raspberry or blackberry seed extract can also be a result of volatiles formation through the oxidation pathway of fatty acids [57], considering that both seeds are rich in fatty acids (Table 2). Accordingly, BBS and RBS aroma can be associated with green, herbal and fruity notes derived from the predominantly present alcohols, aldehydes and esters.

Nevertheless, the investigation of the present VOCs derived aromas contribution to the perception by assessors when including berry powders into food products requires further research, both in detecting major VOCs odour activity values together with the sensory assessment of the berry-enriched product itself.

5-hydroxymethylfurfural, a potentially carcinogenic compound to humans, also formed during heating from dehydration of sugars in acidic environment even at low temperatures [64], was not detected in BBS and RBS, whilst its content was the highest in RB (4.02%), followed by BB (1.19%) and SB (0.64%) (Supplementary Table S2).

### 3.8. ATR-FTIR Spectroscopic Analysis of Berry and Berry Seed Powders

**Figure 3:**
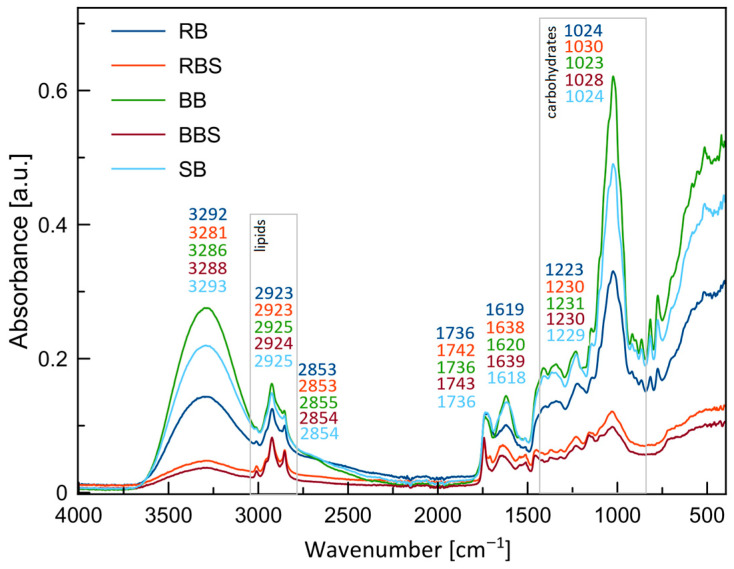
ATR-FTIR spectra of commercial raspberry (RB), raspberry seed (RBS), commercial blackberry (BB), blackberry seed (BBS) and commercial strawberry (SB) powders.

The recorded ATR-FTIR spectra of investigated powders with major detected bands are presented on Figure 3 (Supplementary Table S3). The major regions observed were O–H stretching (centred at ~3300 cm^−1^), C–H stretching (3000–2800 cm^−1^) and the fingerprint region (<1500 cm^−1^), which can be related to bond vibrations in some of the primary constituents present within the samples, namely water, lipids, and carbohydrates, respectively. Additionally, the presence of specific phenolics can be assumed according to the detected vibrations, considering that their content is much lower compared to the previously mentioned compounds.

A strong band centred at 3281–3286 cm^−1^ in samples RBS, BB and BBS and slightly shifted to a higher wavenumber in samples RB and SB (3292 cm^−1^) was assigned to O–H stretching vibrations in bound water, carbohydrates, lignin and flavones as well as intermolecular hydrogen bonds in the corresponding compounds [65,66]. Very weak bands appearing at 3010 cm^−1^ in samples RB, SB and BB, but rather noticeable in RBS and BBS, was related to the =C–H stretching vibration in lipids [67] and was in line with the estimated lipid content of the samples. The subsequent medium bands at ~2924 cm^−1^ and 2854 cm^−1^, visible regardless of sample, are indicative of the CH_2_ asymmetric and symmetric stretching in lipids, respectively, and were previously observed for grape seed [66] and raspberry pomace [11].

The bands appearing at 1742 cm^−1^ in RBS and BBS and slightly shifted to 1736 cm^−1^ in RB, BB and SB were ascribed to the stretching vibrations of the carbonyl group in esterified pectin [66] and simple sugars [11], respectively. Additionally, C=O stretching in lipids is also present within the 1745–1725 cm^−1^ [67].

The medium band at 1638 cm^−1^ in RBS and BBS and shifted medium band at 1619 cm^−1^ in the rest of the samples were attributed to C=O stretching in non-esterified pectin [68]. However, bands in the wavenumber range 1620–1605 cm^−1^ could also be related to C=O stretching in flavonols as well as C=C stretching in aromatic rings of flavonols, flavanols, anthocyanins [65] and ellagitannins [69], which were detected in examined berry and berry seed powders (Figure 1, Supplementary Table S1). Nevertheless, the appearance of amide I band of proteins in this wavenumber range should not be neglected, while the interference of the H–O–H deformation vibration [67] should be minimal since samples were dry. Distinguished very weak bands for spectra of RBS and BBS were recorded at 1544 cm^−1^ corresponding to C=C stretching vibrations as previously found for raspberry pomace [11].

Furthermore, the presence of phenolic compounds is visible through weak bands at 1511 cm^−1^ and 1412–1400 cm^−1^ in berry powders first related to C=C stretching vibrations of A ring and C–H deformation vibrations in flavonols [65], while the second can be connected to C=C stretching vibrations of the ring in anthocyanins [65], but also represent the range of C–H symmetric deformation vibrations of the methyl groups in proteins [70]. Corresponding bands were slightly shifted to higher wavenumbers in berry seed powders (1514 cm^−1^ and 1419 cm^−1^) with the appearance of an additional weak band at 1458 cm^−1^, associated also with deformation vibrations of the methyl groups in proteins and in lipids [70], which content was higher herein (Table 1).

Distinctive bands compared to berry powders (~1342 cm^−1^) were observed in berry seed powders at 1367 and 1315 cm^−1^ for RBS and 1376 cm^−1^ and 1315 cm^−1^ for BBS, associated with CH_2_ out-of-plane and in-plane deformation (scissoring) modes in cellulose and other polysaccharides [66] in line with higher content of TDF in these samples (Table 1). Medium bands in the range 1223–1235 cm^−1^ regardless of sample were assigned to C–O stretching and O–H deformation vibrations in polysaccharides, pectin and phenolic compounds [66]. Moreover, C–O asymmetrical stretching vibrations were noticed through weak bands at ~1156 cm^−1^ in RS, RBS and BBS, as well as those at 1145 cm^−1^ in BB and SB [11], indicative of polysaccharides presence. The strongest absorption bands were detected at ~1030 cm^−1^ for berry seed powders and 1024 cm^−1^ for berry powders, with intensities in descending order BB < SB < RB < RBS < BBS (Figure 3). They were attributed to C–C stretching and C–O stretching and deformation vibration [70] predominantly in cellulose [67]. Moreover, diverse shape of the corresponding bands was observed between berry powders (sharp with the presence of a shoulder) and berry seed powders (broader without shoulder) (Figure 3) implying differences in the TDF content (Table 1). Additionally, the absence of bands at 919 cm^−1^, 866 cm^−1^, 817 cm^−1^ and 776 cm^−1^ was visible in berry seed powders’ spectra compared to those of berry powders. These bands were ascribed to (CCH) and (CCO) deformation vibration in glucose, (CCO) deformation vibration of glucosyl unit in sucrose, (CC) stretching in fructose, and (CCC), (CCO), (OCO) deformation vibration in glucose [71], respectively, which were all present in samples (Table 2). Consequently, they are reflecting the presence of simple sugars which content was significantly higher in berry powders rather than berry seed powders (SB > BB > RB > BBS > RBS, Table 1).

### 3.9. Colour of Berry and Berry Seed Powders

Visually observed colour and results from the instrumental colour analysis of berry and berry seed powders are presented in Table 4. Although statistically different, all flours were characterised by red (a* > 0) and yellowish (b* > 0) colour, with redness most expressed in BBS (21.50), and yellowness most expressed for RBS (32.70, Table 4). Additionally, RBS presented the highest L* values, indicating the lightest colour, followed by BBS, RB, SB and BB, which was the darkest flour sample (Table 4). In addition to the lowest L* values, BB also had the lowest hue angle, chroma, and yellowness, presenting a dark red colour, the most visually distinct compared to other powders (Table 4). The dark red colour of BB could be a consequence of anthocyanins’ presence in the highest quantity in this powder (Table 3) [72], whilst the yellow colouration of RBS and BBS can be attributed to carotenoids [5] and yellow flavonoids [38]. The highest hue angle and chroma values obtained for RBS also imply a shift towards yellow tones and higher colour intensity of this powder [38].

L* values obtained for RB, BB and SB were congruent with results on freeze-dried raspberry, blackberry and strawberry, whilst red and yellowish colouration was reduced (lower a* and b* parameters) [29,50]. Discrepancies were also noted for RBS, characterised by predominantly comparable a*, but higher L* and lower b* values than raspberry seed before and after oil extraction by different methods [38]. The colour of examined powders is another advantage for their further usage as natural colourants in diverse food products where visual appeal is important.

### 3.10. PCA Analysis

**Figure 4:**
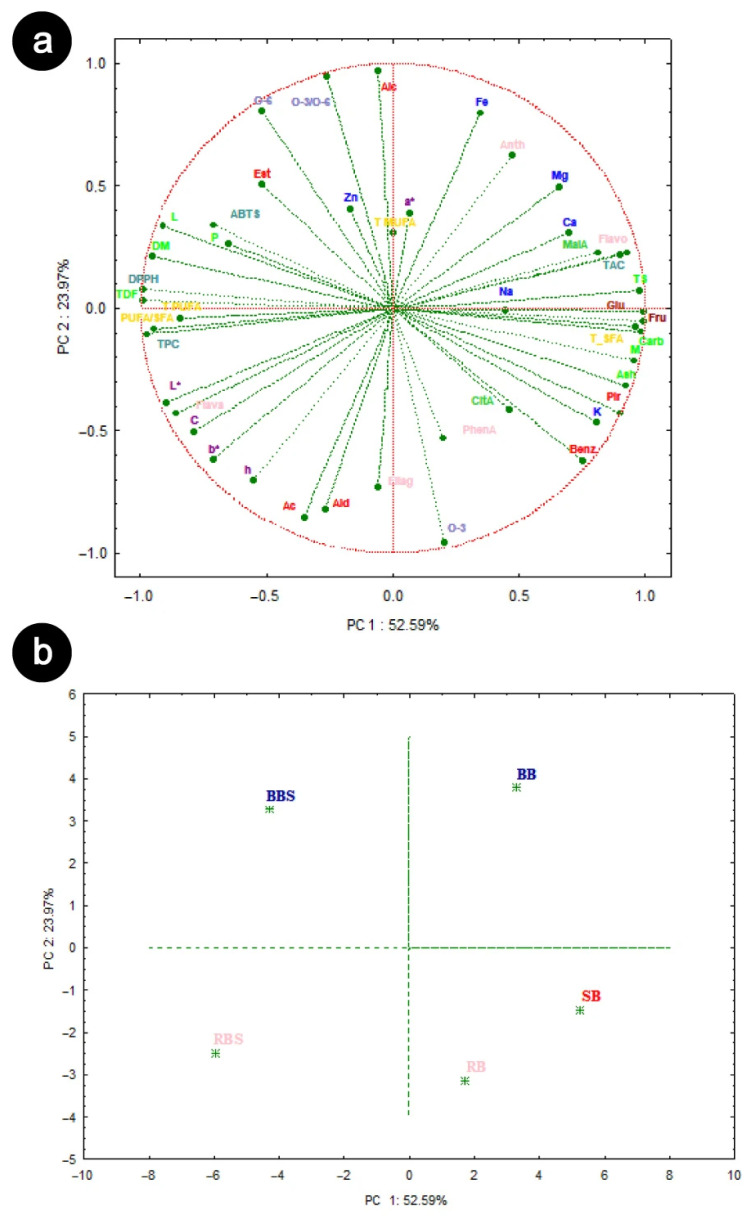
Principle component analysis (PCA) showing loading plot (**a**) and score (**b**) describing the relationship between the berry and berry seed powders and their different characteristics.

Principal components analysis (PCA) is a method for identifying patterns in data and expressing it in a way that highlights similarities and differences [73]. In this study, the PCA was applied to the complete data set (proximate composition, sugars, organic acids, phenols, minerals, fatty acids, volatiles, and antioxidant activity) to determine the most important variables that explain the correlations among three commercial powders (RB, BB, SB) and two powders from seeds (RBS and BBS). Two principal components explained 76.6% of the total variance, with PC1 and PC2 accounting for 52.59% and 23.97%, respectively (Figure 4).

PC1 was positively associated with sugars, mineral composition and pigment-related compounds and negatively with colour parameters. In contrast, the variables that most contributed to PC2 were mainly related to antioxidative activity and fatty acid composition (positive). The score plot revealed a clear separation of samples, indicating pronounced differences in the biochemical profiles of the examined samples. Commercial berry powders were associated with higher values of sugars and mineral elements. In contrast, powders from seeds show closer association with TPC, antioxidant activity, fatty acid composition and colour parameters. The inverse positioning of sugars and phenolic-related variables along PC1 suggests a contrasting pattern between primary metabolites associated with fruit technological quality and secondary metabolites contributing to antioxidant capacity. Similar trends have been reported in other berries, where phenolic accumulation may not coincide with sugar accumulation.

## 4. Conclusions

Consumers’ increased awareness about the importance of nutritionally enhanced food consumption and the environmental effects of its production represents the main driving force behind research aiming towards a more sustainable and circular food chain. In this context, the present study comprehensively evaluates total and individual nutrients’ content, antioxidant and antiradical activity, volatile compounds, colour and functional groups in berry and berry seed powders as a suitable ingredient for further development of added-value food formulations under the umbrella of sustainable and circular economy concepts.

An array of diverse macro and micronutrients was found in investigated powders in quantities exceeding basic nutritional standards, asserting nutritional claims of being “high in” minerals (Mg and Fe) and dietary fibre, allowing the corresponding food product to possibly confer similar claims even when powders are used in small amounts as ingredients. Furthermore, the presented healthy fatty acid profile, characterised by the prevalence of PUFAs and essential omega-3 and omega-6 fatty acids; expression of high antioxidant and antiradical activities; appealing colour; and the green, herbal, floral and fruity aroma of investigated powders, confirms their good potential as food ingredients for achieving a balanced nutritional and a peculiar sensory profile. Nevertheless, among berry powders, SB is distinguished by high sucrose, fructose, omega-3, and mineral (Ca, Mg, Fe) content; BB by superb omega-6, cyanidin-3-*O*-glucoside and quercetin-3-*O*-rutinoside quantities; and RB by citric and chlorogenic acid amounts. Berry seed powders outperform berry powders in TDF, omega-6 fatty acid and individual flavan-3-ols quantity, TPC and activity against DPPH radicals. Highlighted nutritional discrepancies among powders provide a practical implication for the tailored formulation of food products according to nutritional requirements.

Nutritional quality characterisation is the initial step toward recognising the importance of berry and berry seed powders as food ingredients. Taking into account that this is a preliminary study in view of further application in food, further research should comprehensively elucidate the techno-functionality, bioaccessibility and biological activity of displayed nutrients. This will pave the way for the diversification of fruit-enriched food products towards top-notch nutrition, captivating consumers, maximising the health benefits they confer, reducing wastage and supporting the creation of a sustainable and circular food chain.

## Figures and Tables

**Table 2:** Composition of sugars, organic acids and fatty acids in examined berry and berry seed powders.

	RB	RBS	BB	BBS	SB
**Sugars (mg/g)**					
Glucose	123.32 ± 3.10 ^c^	4.45 ± 0.06 ^d^	161.99 ± 3.89 ^b^	9.59 ± 0.28 ^d^	179.25 ± 2.93 ^a^
Fructose	147.82 ± 3.81 ^c^	3.66 ± 0.08 ^d^	189.78 ± 4.90 ^b^	8.39 ± 0.59 ^d^	236.98 ± 4.15 ^a^
Sucrose	68.17 ± 1.63 ^a^	7.11 ± 0.12 ^c^	n.d.	9.33 ± 0.15 ^c^	50.71 ± 1.35 ^b^
Sorbitol	4.48 ± 0.12 ^b^	n.d.	4.89 ± 0.28 ^b^	n.d.	15.63 ± 0.33 ^a^
Sum	343.79	15.22	356.66	27.31	482.57
**Organic acids (mg/g)**					
Citric acid	125.98 ± 2.96 ^a^	35.90 ± 0.50 ^e^	49.42 ± 2.89 ^d^	70.87 ± 3.82 ^c^	86.27 ± 1.10 ^b^
Malic acid	54.44 ± 1.49 ^a^	36.12 ± 1.66 ^d^	52.56 ± 1.71 ^abc^	48.95 ± 0.81 ^bc^	52.82 ± 0.68 ^ab^
Shikimic acid	0.36 ± 0.01 ^c^	0.23 ± 0.01 ^d^	0.48 ± 0.02 ^b^	0.26 ± 0.01 ^d^	0.54 ± 0.01 ^a^
Sum	180.78	72.25	102.46	120.08	139.63
**Fatty acids (g/100 g total fatty acids)**					
Total SFA	10.50 ± 1.05 ^b^	6.00 ± 0.60 ^c^	10.60 ± 1.06 ^b^	7.70 ± 0.77 ^c^	14.20 ± 1.42 ^a^
C12:0	0.1 ± 0.01 ^b^	n.d.	0.2 ± 0.02 ^a^	n.d.	0.1 ± 0.01 ^b^
C14:0	0.3 ± 0.03 ^a^	n.d.	0.3 ± 0.03 ^a^	n.d.	0.2 ± 0.02 ^b^
C16:0	5.20 ± 0.52 ^c^	3.30 ± 0.33 ^d^	6.60 ± 0.66 ^b^	4.20 ± 0.42 ^cd^	9.90 ± 0.99 ^a^
C17:0	0.70 ± 0.07 ^a^	0.10 ± 0.01 ^b^	0.10 ± 0.01 ^b^	0.10 ± 0.01 ^b^	0.10 ± 0.01 ^b^
C18:0	1.50 ± 0.15 ^b^	1.20 ± 0.12 ^b^	2.30 ± 0.23 ^a^	2.20 ± 0.22 ^a^	2.00 ± 0.20 ^a^
C20:0	1.10 ± 0.11 ^a^	0.70 ± 0.07 ^c^	0.90 ± 0.09 ^b^	1.00 ± 0.10 ^ab^	1.00 ± 0.10 ^ab^
C22:0	1.30 ± 0.13 ^a^	0.20 ± 0.02 ^c^	0.20 ± 0.02 ^c^	0.10 ± 0.01 ^c^	0.50 ± 0.05 ^b^
C24:0	0.20 ± 0.02 ^b^	0.50 ± 0.05 ^a^	0.10 ± 0.01 ^c^	0.10 ± 0.01 ^c^	0.20 ± 0.02 ^b^
Total MUFA	16.20 ± 1.62 ^ab^	14.10 ± 1.41 ^b^	14.90 ± 1.49 ^ab^	17.60 ± 1.76 ^a^	15.80 ± 1.58 ^ab^
C16:1	0.30 ± 0.03	0.20 ± 0.02 ^a^	0.20 ± 0.02	0.10 ± 0.01 ^b^	0.30 ± 0.03
C18:1 n-9c	15.80 ± 1.58 ^ab^	13.90 ± 1.39 ^b^	14.60 ± 1.46 ^ab^	17.40 ± 1.74 ^a^	15.50 ± 1.55 ^ab^
Total PUFA	73.30 ± 7.33 ^a^	79.80 ± 7.98 ^a^	74.60 ± 7.46 ^a^	74.70 ± 7.47 ^a^	70.00 ± 7.00 ^a^
C18:2 n-6c	47.30 ± 4.73 ^bc^	54.50 ± 5.54 ^ab^	61.40 ± 6.14 ^a^	62.80 ± 6.28 ^a^	42.70 ± 4.27 ^c^
C18:3 n-3	25.70 ± 2.57 ^a^	25.10 ± 2.51 ^a^	12.90 ± 1.29 ^b^	11.80 ± 1.18 ^b^	27.30 ± 2.73 ^a^
C18:3 n-6	0.10 ± 0.01 ^a^	0.10 ± 0.01 ^a^	n.d.	n.d.	n.d.
C20:2 n-6	n.d.	0.10 ± 0.01 ^a^	0.10 ± 0.01 ^a^	0.10 ± 0.01 ^a^	n.d.
Omega-3	25.70 ± 2.57 ^a^	25.20 ± 2.52 ^a^	13.00 ± 1.30 ^b^	11.80 ± 1.18 ^b^	27.30 ± 2.73 ^a^
Omega-6	47.60 ± 4.76 ^bc^	54.70 ± 5.47 ^ab^	61.50 ± 6.51 ^a^	62.90 ± 6.29 ^a^	42.80 ± 4.28 ^c^
Omega-6/ Omega-3 ratio	1.85 ^cd^	2.17 ^c^	4.73 ^b^	5.33 ^a^	1.57 ^d^
PUFA/SFA ratio	6.98 ^c^	13.3 ^a^	7.04 ^c^	9.70 ^b^	4.93 ^d^

RB—commercial raspberry powder, RBS—raspberry seed powder, BB—commercial blackberry powder, BBS—blackberry seed powder, SB—commercial strawberry powder. SFA—saturated fatty acids, C12:0—lauric acid, C14:0—myristic acid, C16:0—palmitic acid, C17:0—margaric acid, C18:0—stearic acid, C20:0—arachidic acid, C22:0—behenic acid, C24:0—lignoceric acid, MUFA—monounsaturated fatty acids, C16:1—palmitoleic acid, C18:1 n-9c—oleic acid, PUFA—polyunsaturated fatty acids, C18:2 n-6c—linoleic acid, C18:3 n-3—α-linolenic acid, C18:3 n-6—γ-linolenic acid, C20:2 n-6—eicosadienoic acid, n.d.—not detected. The superscript letter means in the rows for each determined parameter followed by different letters are significantly different (*p* < 0.05), according to Duncan’s multiple range test.

**Table 3:** Antioxidant and antiradical activity of examined berry and berry seed powders.

Assay	RB	RBS	BB	BBS	SB
TPC (mg GAE/g)	58.22 ± 4.93 ^b^	77.16 ± 2.34 ^a^	48.05 ± 6.03 ^b^	75.26 ± 2.17 ^a^	50.64 ± 7.84 ^b^
TAC (mg C3G/g)	0.16 ± 0.05 ^b^	n.d.	0.63 ± 0.04 ^a^	n.d.	0.65 ± 0.08 ^a^
DPPH (μmol TE/g)	173.47 ± 1.83 ^b^	229.98 ± 1.56 ^a^	168.68 ± 5.64 ^b^	219.45 ± 6.33 ^a^	141.68 ± 11.96 ^c^
ABTS (μmol TE/g)	19.62 ± 0.47 ^c^	20.67 ± 0.21 ^ab^	20.19 ± 0.15 ^bc^	21.26 ± 0.09 ^a^	13.97 ± 0.45 ^d^

RB—commercial raspberry powder, RBS—raspberry seed powder, BB—commercial blackberry powder, BBS—blackberry seed powder, SB—commercial strawberry powder; total phenolic content—TPC; total anthocyanin content—TAC; DPPH—2,2-diphenyl-1-picrylhydrazyl assay; ABTS—2,2′-azino-bis(3-ethylbenzothiazoline-6-sulfonic acid) radical cation assay. n.d.—not detected. Means in the rows for each determined parameter followed by different letters are significantly different (*p* < 0.05), according to Duncan’s multiple range test.

**Table 4:** Colour parameters and appearance of berry and berry seed powders.

Sample ID	RB	RBS	BB	BBS	SB
L*	40.13 ± 1.10 ^c^	57.53 ± 0.59 ^a^	26.05 ± 0.47 ^e^	48.88 ± 0.65 ^b^	35.53 ± 0.93 ^d^
a*	19.45 ± 0.16 ^b^	11.86 ± 0.15 ^e^	16.24 ± 0.12 ^c^	21.50 ± 0.10 ^a^	15.43 ± 0.32 ^d^
b*	21.72 ± 0.27 ^c^	32.70 ± 0.22 ^a^	10.14 ± 0.09 ^d^	23.51 ± 0.13 ^b^	21.52 ± 0.49 ^c^
*h°*	48.17 ± 0.30 ^c^	70.10 ± 0.19 ^a^	31.99 ± 0.24 ^b^	47.58 ± 0.18 ^c^	54.39 ± 0.38 ^d^
C*					
Appearance	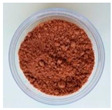	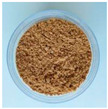	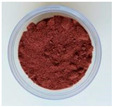	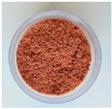	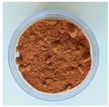

RB—commercial raspberry powder, RBS—raspberry seed powder, BB—commercial blackberry powder, BBS—blackberry seed powder, SB—commercial strawberry powder. L*—brightness (on a lightness-darkness scale), a*—hue on a green (−) to red (+) axis, b*—hue on a blue (−) to yellow (+) axis, *h°*—hue angle, C*—chroma. Means in the rows for each determined parameter followed by different letters are significantly different (*p* < 0.05), according to Duncan’s multiple range test.

## Data Availability

The original contributions presented in this study are included in the article/Supplementary Materials. Further inquiries can be directed to the corresponding author.

## Supplementary Materials

The following supporting information can be downloaded at https://www.mdpi.com/article/10.3390/antiox15060658/s1, Table S1: Profile of individual phenolic compounds in examined berry and berry seed powders. Table S2: Volatile compound (VOC) composition of examined berry and berry seed powders. Table S3: Vibrational band assignments corresponding to the FTIR spectra of berry and berry seed powders. [11,66,68,69,70,71]

## Abbreviations

The following abbreviations are used in this manuscript:

RB	Raspberry flour
BB	Blackberry flour
SB	Strawberry flour
RBS	Raspberry seed flour
BBS	Blackberry seed flour
TDF	Total dietary fibre
PUFA	Polyunsaturated fatty acids
SFA	Saturated fatty acids
TPC	Total phenolic content
DPPH	2,2-difenil-1-pikrilhidrazil
ATR-FTIR	Attenuated total reflectance-Fourier Transform Infrared Spectroscopy
VOCs	Volatile compounds
ABTS	2,2′azynobis(3-etylobenzotiazoline-6-sulfonic acid)
FAME	Fatty acid methyl esters
GAE	Gallic acid equivalent
TAC	Total monomeric anthocyanin content
C3G	Cyanidin-3-O-glucoside equivalents
PCA	Principal components analysis
MUFA	Monounsaturated fatty acids
