# Supplementary material for: Berry Powders as Highly Integrable Food Ingredients: Phenolic and Volatile Compounds Profiling, Comprehensive Nutrient Content Assessment and Spectroscopic Analysis
Source: Antioxidants (Basel). 2026 May 23;15(6):658. doi: 10.3390/antiox15060658 (PMC13295274; doi:10.3390/antiox15060658)
Supplement: Supplementary file 1 [file antioxidants-15-00658-s001.zip › antioxidants-4303683-supplementary.pdf]

Berry powders as highly integrable food ingredients: Phenolic and volatile compounds profiling, comprehensive nutrient content assessment and spectroscopic analysis-Miljana Djordjević, Jelena Tomić, Marijana Djordjević, Aleksandra Bajić, Jelena Živančev, Tilen Zamljen, Jerneja Jakopic

SUPPLEMENTARY MATERIAL

Table S1. Profile of individual phenolic compounds in examined berry and berry seed powders.

**Compounds (mg/kg)**	**RB**	**RBS**	**BB**	**BBS**	**SB**
Phenolic acids	**66.66±9.18[a]**	**4.19±0.42**	**6.74±0.68**	**4.28±1.10**	**9.57±2.09**
Chlorogenic acid	58.18±8.50[a]	n.d.	n.d.	n.d.	n.d.
Ellagic acid	8.48±0.68[ab]	4.19±0.42[c]	6.74±0.68[b]	4.28±1.10[c]	9.57±2.09[a]
Ellagitannins	**17.24±1.75**	**32.39±3.80**	/	**17.81±2.68**	**11.57±0.76**
Ellagitannin	2.69±0.07[c]	17.79±1.32[a]	n.d.	4.71±0.38[b]	n.d.
Ellagic acid pentoside	3.77±0.23[a]	n.d.	n.d.	n.d.	n.d.
Ellagic acid derivative	n.d.	1.69±0.41[b]	n.d.	n.d.	7.64±0.58[a]
Methyl ellagic acid pentoside	2.94±0.22[a]	n.d.	n.d.	n.d.	n.d.
Bis-HHDP-glucose	7.84±1.23[a]	6.21±0.33[b]	n.d.	6.67±0.49[b]	n.d.
Ellagic acid rhamnoside	n.d.	n.d.	n.d.	n.d.	3.93±0.18[a]
Methylellagic acid pentoside derivative 1	n.d.	0.42±0.16[b]	n.d.	1.36±0.44[a]	n.d.
Methylellagic acid pentoside derivative 2	n.d.	0.58±0.42[a]	n.d.	n.d.	n.d.
Ellagic acid pentoside derivative 1	n.d.	3.94±0.45[a]	n.d.	3.67±0.85[a]	n.d.
Ellagic acid pentoside derivative 2	n.d.	1.76±0.71[a]	n.d.	1.40±0.52[a]	n.d.
Flavonols	**8.21±0.61**	/	**19.17±3.3**	**0.57±0.32**	**15.52±4.44**
Quercetin-3-*O*-glucoside	5.71±0.33[a]	n.d.	n.d.	n.d.	6.25±0.85[a]
Quercetin-3,7-di-*O*-hexoside	2.50±0.28[a]	n.d.	n.d.	n.d.	n.d.
Quercetin-3-*O*-pentoside	n.d.	n.d.	2.92±0.30[a]	n.d.	n.d.
Quercetin-3-*O*-rutinoside	n.d.	n.d.	6.26±1.51[a]	n.d.	4.10±1.67[b]
Quercetin-3-*O*-hexoside	n.d.	n.d.	3.65±0.53[a]	n.d.	n.d.
Quercetin-3-*O*-galactoside	n.d.	n.d.	3.47±0.38[a]	n.d.	n.d.
Kaempferol glucuronide	n.d.	n.d.	n.d.	n.d.	2.76±0.62[a]
Kaempferol acetyl hexoside	n.d.	n.d.	n.d.	n.d.	2.41±1.30[a]
Isorhamnetin-3-*O*-hexoronide	n.d.	n.d.	2.87±0.58[a]	n.d.	n.d.
Isorhamnetin glucuronide	n.d.	n.d.	n.d.	0.57±0.32[a]	n.d.

Flavan-3-ols	**9.67±0.66**	**35.26±2.5**	**/**	**11.07±1.02**	**/**
Procyanidin trimer	9.67±0.66[a]	8.47±0.48[b]	n.d.	n.d.	n.d.
Procyanidin B2	n.d.	n.d.	n.d.	11.07±1.02[a]	n.d.
Catechin	n.d.	11.15±0.69[a]	n.d.	n.d.	n.d.
Epicatechin	n.d.	5.86±0.43[a]	n.d.	n.d.	n.d.
Procyanidin dimer	n.d.	9.78±0.90[a]	n.d.	n.d.	n.d.
Anthocyanins	**/**	**/**	**34.72±1.35**	**/**	**4.8±0.91**
Pelargonidin-3-*O*-glucoside	n.d.	n.d.	n.d.	n.d.	4.80±0.91[a]
Cyanidin-3-*O*-glucoside	n.d.	n.d.	34.72±1.35[a]	n.d.	n.d.
SUM	***101.78±12.2***	***71.84±6.72***	***60.63±5.33***	***33.73±5.12***	***41.46±8.2***

Table S2. Volatile compound (VOC) composition of examined berry and berry seed powders.

**Compounds (%)**	**RB**	**RBS**	**BB**	**BBS**	**SB**
Alcohols	*15.78*	*22.76*	*52.94*	*60.68*	*33.01*
1-Hexanol	n.d.	2.32	0.37	3.46	1.78
2-Heptanol	n.d.	n.d.	1.00	2.03	n.d.
2-Propanol, 1-butoxy-	0.04	0.05	n.d.	n.d.	n.d.
1-Heptanol	n.d.	0.07	0.10	0.63	0.40
1-Octen-3-ol	2.89	7.57	2.37	3.23	3.94
1-Hexanol, 2-ethyl-	5.71	n.d.	1.73	1.26	7.78
Benzyl alcohol	0.31	n.d.	n.d.	1.11	0.51
2-Octen-1-ol, (E)-	n.d.	n.d.	n.d.	0.34	n.d.
p-Cresol	0.27	n.d.	0.20	n.d.	0.18
Benzenemethanol, α, α-dimethyl-	0.03	0.09	n.d.	0.10	0.33
2-Furanmethanol, 5-ethenyltetrahydro-α,α,5-trimethyl-, cis-	n.d.	0.35	1.28	n.d.	n.d.
1,6-Octadien-3-ol, 3,7-dimethyl-	3.42	0.10	27.61	0.29	12.37
Phenylethyl Alcohol	1.27	11.76	4.14	45.33	0.86
Bicyclo[3.1.1]heptan-3-ol, 6,6-dimethyl-2-methylene-	n.d.	n.d.	0.14	n.d.	n.d.
3-Nonen-1-ol, (Z)-	n.d.	n.d.	n.d.	0.09	n.d.

Benzenemethanol, α, α,4-trimethyl-	n.d.	n.d.	n.d.	2.46	n.d.
Terpinen-4-ol	n.d.	n.d.	0.31	n.d.	n.d.
3-Cyclohexen-1-ol, 4-methyl-1-(1-methylethyl)-, (R)-	0.15	n.d.	n.d.	0.12	0.37
Benzenemethanol, .alpha.,.alpha.-dimethyl-	0.03	0.09	n.d.	0.10	0.33
L-α-Terpineol	1.23	n.d.	12.92	0.13	n.d.
α-Terpineol	n.d.	n.d.	n.d.	n.d.	3.85
2,6-Octadien-1-ol, 3,7-dimethyl-, (Z)-	n.d.	n.d.	0.61	n.d.	n.d.
Eugenol	0.33	0.36	0.16	n.d.	n.d.
1-Dodecanol	0.04	n.d.	n.d.	n.d.	0.19
1-Hexadecanol	0.06	n.d.	n.d.	n.d.	0.12
Terpens	*2.92*	*1.31*	*4.12*	*0.51*	*3.54*
Bicyclo[2.2.1]heptane, 7,7-dimethyl-2-methylene-	n.d.	n.d.	n.d.	0.09	n.d.
2H-Pyran. 2-ethenyltetrahydro-2.6.6-trimethyl-	n.d.	n.d.	0.15	n.d.	n.d.
7-Oxabicyclo[2.2.1]heptane. 1-methyl-4-(1-methylethyl)-	n.d.	n.d.	0.01	n.d.	n.d.
(+)-4-Carene	n.d.	n.d.	0.20	n.d.	n.d.
o-Cymene	0.22	0.61	0.58	0.42	0.08
Cyclohexene. 1-methyl-5-(1-methylethenyl)-. (R)-	n.d.	0.64	n.d.	n.d.	n.d.
γ-Terpinene	n.d.	n.d.	0.32	n.d.	n.d.
trans-Linalool oxide (furanoid)	2.31	n.d.	n.d.	n.d.	n.d.
Continued **Compounds**	**RB**	**RBS**	**BB**	**BBS**	**SB**
Menthol	0.25	n.d.	n.d.	n.d.	n.d.
Geraniol	n.d.	n.d.	2.86	n.d.	n.d.
Caryophyllene	0.14	0.06	n.d.	n.d.	n.d.
1.6.10-Dodecatrien-3-ol. 3.7.11-trimethyl-. (E)-	n.d.	n.d.	n.d.	n.d.	1.30
2-Furanmethanol. tetrahydro-α,α-5-trimethyl-5-(4-methyl-3- cyclohexen-1-yl)-. [2S-[2-α-5-β (R*)]]-	n.d.	n.d.	n.d.	n.d.	2.16
Norisophrenoids	*2.92*	*0.54*	*0.08*	*0*	*0.27*
α-Ionone	1.14	0.26	n.d.	n.d.	n.d.

trans-β-Ionone	1.78	0.28	0.08	n.d.	0.27
Aldehydes	*29.79*	*39.04*	*18.94*	*12.42*	*22.6*
2-Hexenal, (E)-	0.13	1.40	0.11	0.37	0.08
4-Heptenal, (Z)-	n.d.	0.25	n.d.	n.d.	n.d.
Heptanal	0.34	1.44	0.31	0.14	0.29
2,4-Hexadienal, (E,E)-	n.d.	n.d.	n.d.	0.16	n.d.
2-Heptenal, (Z)-	0.16	1.12	0.07	0.84	0.17
Benzaldehyde	2.12	2.22	1.08	4.18	4.96
2-Furancarboxaldehyde, 5-methyl-	11.54	n.d.	5.60	0.10	5.01
Octanal	1.60	0.40	n.d.	0.37	0.53
2,4-Heptadienal, (E,E)-	n.d.	14.87	n.d.	n.d.	n.d.
1H-Pyrrole-2-carboxaldehyde	0.80	n.d.	0.12	n.d.	2.69
Benzeneacetaldehyde	5.59	1.38	6.63	1.72	6.24
2-Octenal, (E)-	0.46	3.39	0.27	1.30	0.30
Nonanal	0.49	0.54	0.30	0.46	0.86
Lilac aldehyde B	n.d.	n.d.	0.73	n.d.	n.d.
Lilac aldehyde A	n.d.	n.d.	n.d.	n.d.	0.05
2,6-Nonadienal, (E,Z)-	n.d.	4.77	n.d.	0.48	0.43
2,6-Nonadienal, (E,E)-	n.d.	n.d.	0.33	n.d.	n.d.
2-Nonenal, (E)-	0.53	2.56	0.38	1.00	0.35
Lilac aldehyde D	n.d.	n.d.	0.47	n.d.	n.d.
cis-4-Decenal	n.d.	0.22	n.d.	n.d.	n.d.
1,3-Cyclohexadiene-1-carboxaldehyde, 2,6,6-trimethyl-	1.09	n.d.	0.31	n.d.	n.d.
Decanal	0.21	0.16	n.d.	n.d.	n.d.
2,4-Nonadienal, (E,E)-	n.d.	2.80	n.d.	0.32	n.d.
1-Cyclohexene-1-carboxaldehyde, 2,6,6-trimethyl-	0.20	n.d.	0.28	n.d.	n.d.
5-Hydroxymethylfurfural	4.02	n.d.	1.19	n.d.	0.64
Cinnamaldehyde, (E)-	n.d.	n.d.	n.d.	0.04	n.d.

Continued **Compounds**	**RB**	**RBS**	**BB**	**BBS**	**SB**
Benzeneacetaldehyde, α-ethylidene-	n.d.	n.d.	0.26	0.10	n.d.
2,4-Decadienal, (E,E)-	n.d.	1.45	n.d.	n.d.	n.d.
2-Octenal, 2-butyl-	n.d.	0.03	n.d.	n.d.	n.d.
Vanillin	0.45	0.04	0.48	0.84	n.d.
Dodecanal	n.d.	n.d.	0.02	n.d.	n.d.
Tetradecanal	0.03	n.d.	n.d.	n.d.	n.d.
Pentadecanal	0.03	n.d.	n.d.	n.d.	n.d.
Acids	*8.79*	*8.68*	*1.45*	*1.96*	*2.84*
Butanoic acid, 3-methyl-	2.82	n.d.	n.d.	n.d.	n.d.
Butanoic acid, 2-methyl-	n.d.	5.98	n.d.	n.d.	n.d.
Pentanoic acid	n.d.	2.37	0.50	0.41	0.42
Pentanoic acid, 4-oxo-	0.48	n.d.	n.d.	n.d.	n.d.
Heptanoic acid	n.d.	n.d.	n.d.	0.81	0.05
Hexanoic acid, 2-ethyl-	n.d.	n.d.	n.d.	0.12	n.d.
Octanoic acid	3.92	n.d.	n.d.	0.47	0.39
Benzeneacetic acid	n.d.	0.26	n.d.	0.10	n.d.
Nonanoic acid	1.26	0.02	0.76	n.d.	1.93
Dodecanoic acid	0.13	n.d.	0.13	n.d.	n.d.
Tetradecanoic acid	0.05	n.d.	0.03	n.d.	n.d.
n-Hexadecanoic acid	0.13	0.05	0.03	0.05	0.05
Esters	*3.23*	*4.29*	*2.45*	*25.25*	*4.08*
Propanoic acid, 2-hydroxy-, ethyl ester	n.d.	n.d.	0.32	n.d.	n.d.
Butanoic acid, ethyl ester	n.d.	n.d.	0.06	0.15	n.d.
Butanoic acid, 3-methyl-, ethyl ester	n.d.	0.10	n.d.	n.d.	n.d.
1-Butanol, 3-methyl-, acetate	n.d.	0.38	n.d.	5.21	0.08
1-Butanol, 2-methyl-, acetate	0.04	0.24	n.d.	2.06	n.d.

Acetic acid, pentyl ester	n.d.	0.09	n.d.	n.d.	n.d.
Hexanoic acid, methyl ester	0.41	0.28	0.22	0.06	0.51
Ethyl tiglate	n.d.	0.08	n.d.	n.d.	n.d.
2-Furanmethanol, acetate	0.14	n.d.	0.06	n.d.	n.d.
Hexanoic acid, ethyl ester	n.d.	n.d.	n.d.	0.41	n.d.
Acetic acid, hexyl ester	n.d.	0.04	n.d.	0.22	0.04
2,3-Butanediol, diacetate	n.d.	n.d.	n.d.	1.65	n.d.
2-Butenoic acid, 2-methyl-, 2-methylpropyl ester	n.d.	0.03	n.d.	n.d.	n.d.
Ethyl 3-acetoxybutyrate	n.d.	n.d.	n.d.	0.59	n.d.
Octanoic acid, methyl ester	n.d.	0.09	n.d.	n.d.	n.d.
Acetic acid, phenylmethyl ester	0.34	n.d.	n.d.	0.24	0.59

Continued **Compounds**	**RB**	**RBS**	**BB**	**BBS**	**SB**
Benzeneacetic acid, methyl ester	n.d.	0.97	n.d.	n.d.	n.d.
Methyl salicylate	n.d.	0.29	n.d.	n.d.	n.d.
Octanoic acid, ethyl ester	n.d.	0.02	n.d.	n.d.	n.d.
Acetic acid, 2-phenylethyl ester	n.d.	0.59	n.d.	8.70	n.d.
Bicyclo[2.2.1]heptan-2-ol, 1,7,7-trimethyl-, acetate, (1S-endo)-	n.d.	0.02	n.d.	n.d.	n.d.
Isobornyl acetate	n.d.	n.d.	n.d.	0.05	n.d.
Nonanoic acid, 9-oxo-, methyl ester	n.d.	n.d.	n.d.	n.d.	0.10
Diethyl Phthalate	0.62	n.d.	0.47	0.03	0.83
Dodecanoic acid, ethyl ester	0.32	n.d.	n.d.	n.d.	n.d.
Dodecanoic acid, 1-methylethyl ester	n.d.	n.d.	n.d.	n.d.	0.04
Methyl tetradecanoate	n.d.	n.d.	0.07	n.d.	n.d.
Tetradecanoic acid, ethyl ester	n.d.	n.d.	0.05	n.d.	n.d.
Isopropyl myristate	0.06	n.d.	n.d.	n.d.	0.08
1,2-Benzenedicarboxylic acid, bis(2-methylpropyl) ester	n.d.	0.04	n.d.	n.d.	0.26
Hexadecanoic acid, methyl ester	0.28	0.07	0.35	n.d.	0.56
Dibutyl phthalate	0.38	n.d.	n.d.	n.d.	0.40

Hexadecanoic acid, ethyl ester	0.09	0.03	0.19	0.05	n.d.
Propanoic acid, 2-hydroxy-, ethyl ester	n.d.	n.d.	0.32	n.d.	n.d.
Butanoic acid, ethyl ester	n.d.	n.d.	0.06	0.15	n.d.
Butanoic acid, 3-methyl-, ethyl ester	n.d.	0.10	n.d.	n.d.	n.d.
1-Butanol, 3-methyl-, acetate	n.d.	0.38	n.d.	5.21	0.08
Acetic acid, pentyl ester	n.d.	0.09	n.d.	n.d.	n.d.
Hexanoic acid, methyl ester	0.41	0.28	0.22	0.06	0.51
Ethyl tiglate	n.d.	0.08	n.d.	n.d.	n.d.
2-Furanmethanol, acetate	0.14	n.d.	0.06	n.d.	n.d.
Hexanoic acid, ethyl ester	n.d.	n.d.	n.d.	0.41	n.d.
Ketons	*18.95*	*6.86*	*9.63*	*3.04*	*16.46*
3-Heptanone	n.d.	0.03	n.d.	n.d.	n.d.
2-Heptanone	0.31	2.62	0.22	0.86	n.d.
Ethanone, 1-(2-furanyl)-	4.24	n.d.	3.68	0.03	7.91
2,3-Octanedione	n.d.	n.d.	0.07	n.d.	0.27
5-Hepten-2-one, 6-methyl-	2.02	0.39	1.03	0.27	0.44
2-Octanone	n.d.	0.36	n.d.	n.d.	n.d.
3-Octen-2-one	0.50	0.99	n.d.	0.46	n.d.
Acetophenone	1.22	0.86	0.66	0.92	3.25
Ethanone, 1-(1H-pyrrol-2-yl)-	6.60	n.d.	3.07	n.d.	3.86
3,5-Octadien-2-one	1.15	1.58	n.d.	0.39	n.d.
Continued **Compounds**	**RB**	**RBS**	**BB**	**BBS**	**SB**
Maltol	0.46	n.d.	n.d.	n.d.	n.d.
4-Acetyl-1-methylcyclohexene	n.d.	n.d.	0.16	n.d.	0.13
Camphor	0.03	n.d.	n.d.	n.d.	n.d.
Pinocarvone	n.d.	n.d.	0.20	n.d.	n.d.
2-Buten-1-one, 1-(2,6,6-trimethyl-1,3-cyclohexadien-1-yl)-, (E)-	0.44	n.d.	0.33	0.06	0.34
5,9-Undecadien-2-one, 6,10-dimethyl-, (E)-	0.03	n.d.	n.d.	n.d.	n.d.

2,5-Cyclohexadiene-1,4-dione, 2,6-bis(1,1-dimethylethyl)-	0.76	n.d.	0.21	0.05	0.08
4-(2,6,6-Trimethylcyclohexa-1,3-dienyl)but-3-en-2-one	1.05	n.d.	n.d.	n.d.	0.18
2-Butanone, 4-(4-hydroxyphenyl)-	0.11	n.d.	n.d.	n.d.	n.d.
2-Butanone, 4-(4-hydroxy-3-methoxyphenyl)-	0.03	n.d.	n.d.	n.d.	n.d.
3-Heptanone	n.d.	0.03	n.d.	n.d.	n.d.
Benzens	*0.25*	*0.21*	*0.04*	*0*	*0*
Ethylbenzene	0.11	0.15	0.04	n.d.	n.d.
Caryophyllene	0.14	0.06	n.d.	n.d.	n.d.
Furans	*11.95*	*3.05*	*8.17*	*0.83*	*13.79*
2(3H)-Furanone, 5-methyl-	8.76	n.d.	6.46	n.d.	4.70
2-n-Butyl furan	n.d.	0.24	n.d.	n.d.	n.d.
2-Acetyl-5-methylfuran	0.16	n.d.	0.20	n.d.	n.d.
Furan, 2-pentyl-	0.03	1.06	n.d.	0.37	n.d.
2(3H)-Furanone, 5-ethyldihydro-	n.d.	n.d.	n.d.	n.d.	0.25
2(3H)-Furanone, 5-hexyldihydro-	n.d.	n.d.	n.d.	n.d.	7.34
2(4H)-Benzofuranone, 5,6,7,7a-tetrahydro-4,4,7a-trimethyl-, (R)-	0.24	n.d.	0.02	n.d.	n.d.
5-Acetoxymethyl-2-furaldehyde	0.99	n.d.	0.21	n.d.	n.d.
2(5H)-Furanone, 5-methyl-	1.47	n.d.	0.93	n.d.	0.93
2(3H)-Furanone, dihydro-5-pentyl-	0.30	1.75	0.35	0.46	0.57
Alkanes	*0*	*0.04*	*1.89*	*0*	*0*
Heptane, 2,2,4,6,6-pentamethyl-	n.d.	n.d.	1.89	n.d.	n.d.
Tetradecane, 5-methyl-	n.d.	0.04	n.d.	n.d.	n.d.
Pirazines	*0*	*0*	*0*	*0.14*	*0.12*
Pyrazine, 2-ethyl-6-methyl-	n.d.	n.d.	n.d.	n.d.	0.12
Pyrazine, 3-ethyl-2,5-dimethyl-	n.d.	n.d.	n.d.	0.14	n.d.

RB-commercial raspberry powder; RBS-raspberry seed powder; BB-commercial blackberry powder; BBS-blackberry seed powder; SB-commercial strawberry powder; n.d.-not detected.

Table S3. Vibrational band assignments corresponding to the FTIR spectra of berry and berry seed powders.

RB	RBS	BB	BBS	SB	Band assignment	Reference
3292				3293	ν(O-H), stretching vibration mainly in water	Różyło et al., 2021
	3281	3286	3288		ν(O-H), stretching vibrations-polysaccharides, lignins, flavones	Nogales-Bueno et al., 2017a; Krysa, Szymańska-Chargot & Zdunek, 2022
3011	3010		3010	3014	=C-H stretching vibration-lipids	Stuart, 2004
2923	2923	2925	2924	2925	$\nu_a(CH_2)$, asymmetric stretching-lignins, lipids<br>$\nu_a(CH_2)$, asymmetric stretching-phytochemical species such as tannic acid	Nogales-Bueno et al., 2017a;<br>Patle et al., 2020
2853	2853	2855	2854	2854	$\nu_s(CH_2)$, symmetric stretching waxes, cutin, lipids<br>ν(C-H) in CH_2 and CH_3, symmetric stretching vibrations	Nogales-Bueno et al., 2017a;<br>Różyło et al., 2023
	1742		1743		ν(C=O)ester, stretching vibrations-polyesters, pectins, lignins<br>ν(C=O), stretching vibrations-simple sugars	Nogales-Bueno et al., 2017a; Różyło et al., 2023; Stuart, 2004
1736		1736		1736	ν(C=O)ester, stretching vibrations-polyesters, pectins, lignins,<br>ν(C=O), stretching vibrations-simple sugars	Nogales-Bueno et al., 2017a; Różyło et al., 2023; Stuart, 2004
	1638		1639		ν(C=O), stretching in non-esterified pectin	Nogales-Bueno et al., 2017b
1619		1620		1618	ν(C=O), stretching vibrations in non-esterified pectin, flavonols<br>ν(C=C), stretching vibrations in aromatic rings of flavonols, flavanols and anthocyanins<br>ν(C=C), stretching vibrations in aromatic rings of ellagitannins	Nogales-Bueno et al., 2017b; Krysa, Szymańska-Chargot & Zdunek, 2022;<br>Basalekou et al., 2019
	1544		1543		ν(C=C), stretching vibrations	Różyło et al., 2023
1511	1511	1514	1510	1514	ν(C=C), stretching vibrations of A ring<br>β(C−H), deformation vibrations flavonols	Krysa, Szymańska-Chargot & Zdunek, 2022
		1412		1411	ν(C=C), stretching vibrations of the ring in anthocyanins<br>β(C-H), symmetric deformation vibrations of the methyl groups in proteins	Krysa, Szymańska-Chargot & Zdunek, 2022;

RB	RBS	BB	BBS	SB	Band assignment	Reference
						Movasaghi, Rehman, & ur Rehman, 2008
		1376			β(CH_2) out of plane, out of plane deformation vibration-cellulose, pectin	Nogales-Bueno et al., 2017a
	1367				β(CH_2) out of plane, out of plane deformation vibration-cellulose, pectin	Nogales-Bueno et al., 2017a
**RB**	**RBS**	**BB**	**BBS**	**SB**	**Band assignment**	**Reference**
1341		1342		1343	β(CH_2) out of plane and in-plane deformation vibrations	/
	1315		1315		β(CH_2) in-plane deformation-cellulose, pectin	Nogales-Bueno et al., 2017a
	1230	1231	1230	1229	ν(C-O), stretching vibrations-polysaccharides, pectin and phenolic compounds<br>β(O-H), deformation vibrations-polysaccharides, pectin and phenolic compounds	Nogales-Bueno et al., 2017a
1223					ν(C-O), stretching vibrations-polysaccharides, pectin and phenolic compounds	Nogales-Bueno et al., 2017a
1155	1156		1157		ν_a(C-O), asymmetrical stretching vibrations-polysaccharides	Różyło et al., 2023
		1145		1143	ν_a(C-O), asymmetrical stretching vibrations-polysaccharides	Różyło et al., 2023
**1024**	**1030**	**1023**	**1028**	**1024**	ν(C-C), stretching vibrations-cellulose<br>ν(C-O), stretching vibrations-cellulose<br>β(C-O), deformation vibration-cellulose	Movasaghi, Rehman, & ur Rehman, 2008;<br>Stuart, 2004
919		917		919	β(CCH), deformation vibrations-glucose<br>β(CCO), deformation vibrations-glucose	Wiercigroch et al., 2017
866		865		865	β(CCO), deformation vibrations-glucosyl unit in succrose	Wiercigroch et al., 2017
816		817		817	ν(CC), stretching vibrations-fructose	Wiercigroch et al., 2017
775		776		776	β(CCC), deformation vibrations-glucose<br>β(CCO), deformation vibrations-glucose<br>β(OCO), deformation vibrations-glucose	Wiercigroch et al., 2017

RB-commercial raspberry powder; RBS-raspberry seed powder; BB-commercial blackberry powder; BBS-blackberry seed powder; SB-commercial strawberry powder.

ν-stretching vibration; β-deformation (bending) vibration.

bold-very strong band-maximum band

References

Basalekou, M., Kallithraka, S., Tarantilis, P. A., Kotseridis,Y., & Pappas, C. (2019). Ellagitannins in wines: Future prospects in methods of analysis using FT-IR spectroscopy. *LWT - Food Science and Technology*, 101, 48–53. https://doi.org/10.1016/j.lwt.2018.11.017.

Krysa, M., Szymańska-Chargot, M., & Zdunek, A. (2022). FT-IR and FT-Raman fingerprints of flavonoids – A review. *Food Chemistry*, 393, 133430. https://doi.org/10.1016/j.foodchem.2022.133430.

Movasaghi, Z., Rehman, S., & ur Rehman, I. (2008). Fourier Transform Infrared (FTIR) Spectroscopy of Biological Tissues. *Applied Spectroscopy Reviews*, *43*(2), 134–179. https://doi.org/10.1080/05704920701829043.

Nogales-Bueno, J., Baca-Bocanegra, B., Rooney, A., Hernández-Hierro, J. M., Heredia, F. J., & Byrne, H. J. (2017b). Linking ATR-FTIR and Raman features to phenolic extractability and other attributes in grape skin. *Talanta, 167*, 44–50, https://doi.org/10.1016/j.talanta.2017.02.008.

Nogales-Bueno, J., Baca-Bocanegra, B., Rooney, A., Hernández-Hierro, J. M., Byrne, H. J., & Heredia, F. J. (2017a). Study of phenolic extractability in grape seeds by means of ATR-FTIR and Raman spectroscopy. *Food Chemistry*, *232*, 602–609. http://dx.doi.org/10.1016/j.foodchem.2017.04.049.

Różyło, R., Amarowicz, R., Janiak, M. A., Domin, M., Gawłowski, S., Kulig, R., Łysiak, G., Rząd, K., & Matwijczuk, A. (2023). Micronized Powder of Raspberry Pomace as a Source of Bioactive Compounds. *Molecules*, *28*, 4871. https://doi.org/10.3390/molecules28124871.

Wiercigroch, E., Szafraniec, E., Czamara, K., Pacia, M. Z., Majzner, K., Kochan, K., Kaczor, A., Baranska, M., & Malek, K. (2017). Raman and infrared spectroscopy of carbohydrates: A review. *Spectrochimica Acta Part A: Molecular and Biomolecular Spectroscopy, 185,* 317–335. https://doi.org/10.1016/j.saa.2017.05.045.
